# Supplementary material for: Study protocol for a comparative effectiveness evaluation of abiraterone acetate against enzalutamide: a longitudinal study based on Swedish administrative registers
Source: BMJ Open. 2021 Oct 22;11(10):e052610. doi: 10.1136/bmjopen-2021-052610 (PMC8547362; doi:10.1136/bmjopen-2021-052610)
Supplement: Supplementary data [file bmjopen-2021-052610supp001.pdf]

Figure S1: The fraction (the number of diagnoses divided by the number of men in the population) with a prostate cancer diagnose.

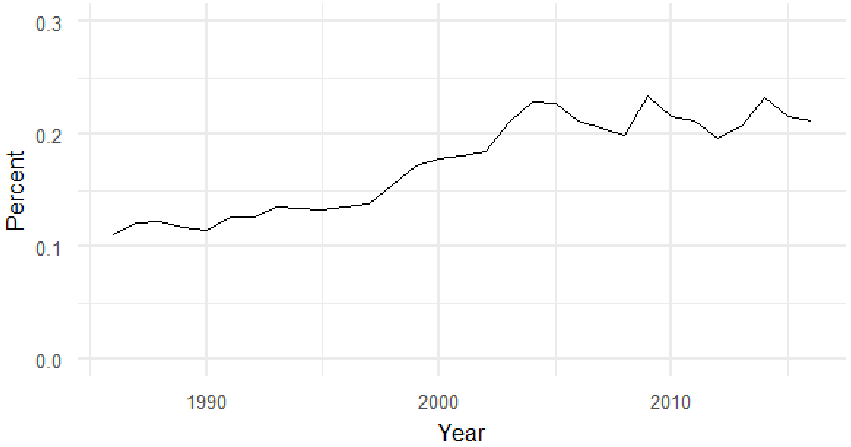

Table S1: Number of collected first time prescriptions per year after the subsidization, by year of prescription.

Year	Enzalutamide	Abiraterone	Total
2014	0	2	2
2015	864	407	1271
2016	1158	233	1391
2017	1005	321	1326
2018	454	157	611
Total	3481	1120	4601

Note: The reason for the drop in 2018 is that data covers only the period up to June 15 2018.

Table S2: ICD codes used to specify related diagnoses

Cardiovascular disease	Diabetes	Osteoporosis	Metastases	Malaise and fatigue
I21	E10	M859	C77	R53
I22	E11	M810	C78	
I252	E12	M818	C79	
Z958	E13	M819		
Z959	E14			
I70				
I71				

I731<br>I738<br>I739<br>I771<br>I790<br>I792<br>K551<br>K558<br>K559<br>I090<br>I110<br>I13<br>I130<br>I131<br>I132<br>I139<br>I42<br>I420<br>I421<br>I422<br>I423<br>I424<br>I425<br>I426<br>I427<br>I428<br>I429<br>I43<br>I44<br>I45<br>I46<br>I47<br>I48<br>I49<br>I50<br>I51<br>R00				

Table S3: All variables with description

	**Variable**	**Description**
	LOPNR	ID serial number
	group	Treatment group (group=1 for abiraterone)
	lan_namn	County council
	h_tot_ov_bfd_i	Indicator of other CVD before diagnosis
	h_tot_ov_bw_i	Indicator of other CVD between diagnosis and treatment
	d_tot_bfd_i	Indicator of diabetes before diagnosis
	d_tot_bw_i	Indicator of diabetes between diagnosis and treatment
	o_tot_bfd_i	Indicator of osteoporosis before diagnosis
	o_tot_bw_i	Indicator of osteoporosis between diagnosis and treatment
	c_tot_bfd_i	Indicator of metastases before diagnosis
	c_tot_bw_i	Indicator of metastases between diagnosis and treatment
	f_tot_bfd_i	Indicator of fatigue before diagnosis
	f_tot_bw_i	Indicator of fatigue between diagnosis and treatment
	h_tot_ov_i	Indicator of other CVD before treatment
	d_tot	Indicator of diabetes before treatment
	o_tot	Indicator of osteoporosis before treatment
	c_tot	Indicator of metastases before treatment
	i48	Indicator of atrial fibrillation and flutter before treatment
	i21	Indicator of acute myocardial infarction before treatment
	i48_bfd_i	Indicator of atrial fibrillation and flutter before diagnosis
	i21_bfd_i	Indicator of acute myocardial infarction before diagnosis
	i48_bw_i	Indicator of atrial fibrillation and flutter before diagnosis
	i21_bw_i	Indicator of acute myocardial infarction before diagnosis
	f_tot	Indicator of fatigue before treatment
	h_tot	Indicator of any CVD before treatment
	Diff_time	Time between diagnosis and treatment
‘	bfd_1m	Number of visits, 1 month before diagnosis
‘	bfd_12m	Number of visits, 12 months before diagnosis
‘	bfd_60m	Number of visits, 5 years before diagnosis
‘	afd_1m	Number of visits, 0-1 month after diagnosis
‘	afd_2m	Number of visits, 1-2 months after diagnosis
‘	afd_3m	Number of visits, 2-3 months after diagnosis
‘	bft_1m	Number of visits, 0-1 month before treatment
‘	bft_2m	Number of visits, 1-2 months before treatment
‘	bft_3m	Number of visits, 2-3 months before treatment
‘	bft_4m	Number of visits, 3-4 months before treatment
‘	bft_5m	Number of visits, 4-5 months before treatment
‘	bft_12m_tot	Number of visits, 1 year before treatment
‘	bft_12m_tot_c619	Number of C61.9 related visits, 1 year before treatment
‘	h_bfd_12m	Number of heart related visits, 1 year before treatment

‘	h_bft_3m	Number of heart related visits, 3 months before treatment
‘	msv_days_bd	Number of days in inpatient care before diagnosis
‘	msv_days_c619	Number of days in inpatient care related to C61.9
‘	msv_tot_prop_c619	Fraction of days in inpatient care related to C61.9
‘	msv_days	Days in inpatient care between diagnosis and treatment
‘	msv_tot_prop	Fraction of days in inpatient care between diagnosis and treatment
‘	nn	Visits per day between diagnosis and treatment
	h_tot_bfd	Visits related to CVD before diagnosis
	h_tot_bw	Visits related to CVD between diagnosis and treatment
‘	d_tot_bfd	Visits related to diabetes before diagnosis
‘	d_tot_bw	Visits related to diabetes between diagnosis and treatment
‘	o_tot_bfd	Visits related to osteoporosis before diagnosis
‘	o_tot_bw	Visits related to osteoporosis between diagnosis and treatment
‘	c_tot_bfd	Visits related to metastases before diagnosis
‘	c_tot_bw	Visits related to metastases between diagnosis and treatment
‘	f_tot_bfd	Visits related to fatigue before diagnosis
‘	f_tot_bw	Visits related to fatigue between diagnosis and treatment
‘	i48_bfd	Visits related to atrial fibrillation and flutter before diagnosis
‘	i21_bfd	Visits related to acute myocardial infarction before diagnosis
‘	h_tot_ov_bfd	Visits related to other CVD before diagnosis
‘	i48_bw	Visits related to atrial fibrillation and flutter between diagnosis and treatment
‘	i21_bw	Visits related to acute myocardial infarction between diagnosis and treatment
‘	h_tot_ov_bw	Visits related to other CVD between diagnosis and treatment
‘	q1_t	Number of inpatient care visits between diagnosis and treatment, quartile 1
‘	q2_t	Number of inpatient care visits between diagnosis and treatment, quartile 2
‘	q3_t	Number of inpatient care visits between diagnosis and treatment, quartile 3
‘	q4_t	Number of inpatient care visits between diagnosis and treatment, quartile 4
‘	n_heart_kalc	Number of prescriptions of ATC code C08
‘	n_heart_beta	Number of prescriptions of ATC code C07
‘	n_diab	Number of prescriptions of ATC code A10
	Mort_medel	Historical county specific mortality in prostate cancer
	Fodelseland_EU28	Country of birth
	ALDER	Age at treatment
	Utbn=ForGymn	Educational level at treatment : less than secondary school
	Utbn=Gymn	Educational levelat treatment : secondary school
	Utbn=EfterGymn	Educational level at treatment : more than secondary school
	Civil	Marital status at treatment (Civil=1 if partner)
‘	LoneInk.x	Wage income at treatment

’	InkFNetto	Income from business at treatment
‘	ForvErs	Wage income at treatment
’	KapInk	Capital income at treatment
’	DispInk04	Disposable income at treatment
’	DispInkFam04	Family disposable income at treatment
‘	ArbErs	Wage income at treatment
’	LoneInk_1y	Wage income one year before treatment
‘	InkFNetto_1y	Income from business one year before treatment
‘	KapInk_1y	Capital income one year before treatment
‘	DispInk04_1y	Disposable income one years before treatment
‘	DispInkFam04_1y	Family disposable income one years before treatment
‘	ForvErs_1y	Wage income one year before treatment
‘	LoneInk_2y	Wage income two years before treatment
‘	InkFNetto_2y	Income from business two years before treatment
‘	ForvErs_2y	Wage income two years before treatment
‘	KapInk_2y	Capital income two years before treatment
‘	DispInk04_2y	Disposable income two years before treatment
‘	DispInkFam04_2y	Family disposable income two years before treatment
‘	SjukRe	Sickness compensation at treatment
’	ArbLos	Unemployment benefits at treatment
’	ForTid	Early retirement benefit at treatment
‘	SocInk	Social security benefits at treatment
‘	SocBidrPersF04	Social security benefits at treatment
‘	SocBidrFam	Social security benefits of the family at treatment
‘	SjukRe_1y	Sickness compensation one year before treatment
‘	ArbLos_1y	Unemployment benefits one year before treatment
‘	ForTid_1y	Early retirement benefit one year before treatment
‘	SocInk_1y	Social security benefits one year before treatment
‘	SocBidrPersF04_1y	Social security benefits one year before treatment
‘	SocBidrFam_1y	Social security benefits of the family one year before treatment
‘	SjukRe_2y	Sickness compensation two years before treatment
‘	ArbLos_2y	Unemployment benefits two years before treatment
‘	ForTid_2y	Early retirement benefit two years before treatment
‘	SocInk_2y	Social security benefits two years before treatment
‘	SocBidrPersF04_2y	Social security benefits two years before treatment
‘	SocBidrFam_2y	Social security benefits of the family two years before treatment
‘	SumAldP03	Old-age pensions at treatment
‘	SumTjP	Occupational pensions one year before treatment
‘	PrivPens	Private pensions two years before treatment
‘	SumAldP03_1y	Old-age pensions at treatment
‘	SumTjP_1y	Occupational pensions one year before treatment
‘	PrivPens_1y	Private pensions two years before treatment
‘	SumAldP03_2y	Old-age pensions at treatment
‘	SumTjP_2y	Occupational pensions one year before treatment

‘	PrivPens_2y	Private pensions two years before treatment
	ALDER_D	Age at diagnosis
	Civil_D	Marital status at diagnosis (Civil=1 if partner)
‘	LoneInk_D	Wage income at diagnosis
’	InkFNetto_D	Income from business at diagnosis
’	KapInk_D	Capital income at diagnosis
’	DispInk_D	Disposable income at diagnosis
’	DispInkFam_D	Family disposable income at diagnosis
’	LoneInk_1y_D	Wage income one year before diagnosis
‘	InkFNetto_1y_D	Income from business one year before diagnosis
‘	KapInk_1y_D	Capital income one year before diagnosis
‘	DispInk_1y_D	Disposable income one year before diagnosis
‘	DispInkFam_1y_D	Family disposable income one year before diagnosis
‘	LoneInk_2y_D	Wage income two years before diagnosis
‘	InkFNetto_2y_D	Income from business two years before diagnosis
‘	KapInk_2y_D	Capital income two years before diagnosis
‘	DispInk_2y_D	Disposable two years before diagnosis
‘	DispInkFam_2y_D	Family disposable income two years before diagnosis
‘	SjukRe_D	Sickness compensation at diagnosis
’	ArbLos_D	Unemployment benefits at diagnosis
’	ForTid_D	Early retirement benefit at diagnosis
‘	SocInk_D	Social security benefits at diagnosis
‘	SocBidrPersF_D	Social security benefits at diagnosis
‘	SocBidrFam_D	Social security benefits of the family at diagnosis
‘	SjukRe_1y_D	Sickness compensation one year before diagnosis
‘	ArbLos_1y_D	Unemployment benefits one year before diagnosis
‘	ForTid_1y_D	Early retirement benefit one year before diagnosis
‘	SocInk_1y_D	Social security benefits one year before diagnosis
‘	SocBidrPersF_1y_D	Social security benefits one year before diagnosis
‘	SocBidrFam_1y_D	Social security benefits of the family one year before diagnosis
‘	SjukRe_2y_D	Sickness compensation two years before diagnosis
‘	ArbLos_2y_D	Unemployment benefits two years before diagnosis
‘	ForTid_2y_D	Early retirement benefit two years before diagnosis
‘	SocInk_2y_D	Social security benefits two years before diagnosis
‘	SocBidrPersF_2y_D	Social security benefits two years before diagnosis
‘	SocBidrFam_2y_D	Social security benefits of the family two years before diagnosis
‘	AldPens_D	Old-age pensions at diagnosis
‘	SumTjP_D	Occupational pensions one year before diagnosis
‘	PrivPens_D	Private pensions two years before diagnosis
‘	AldPens_1y_D	Old-age pensions at diagnosis
‘	SumTjP_1y_D	Occupational pensions one year before diagnosis
‘	PrivPens_1y_D	Private pensions two years before diagnosis
‘	AldPens_2y_D	Old-age pensions at diagnosis
‘	SumTjP_2y_D	Occupational pensions one year before diagnosis

‘	PrivPens_2y_D	Private pensions two years before diagnosis

Table S4: Estimated factor loadings

	F1	F2	F3	F4	F5	F6	F7	F8	F9
SocBidrPers_1y_D	0.94								
SocBidrPers_D	0.93								
SocBidrFam_D	0.92								
SocBidrPers_2y_D	0.91								
SocBidrFam_2y_D	0.90								
SocBidrFam_1y_D	0.90								
SocBidrPersF04_2y	0.88								
SocBidrFam_2y	0.87								
SocBidrPersF04_1y	0.87								
SocBidrFam	0.86								
SocBidrPersF04	0.85								
SocBidrFam_1y	0.84								
LoneInk_1y		0.95	0.21						
LoneInk.x		0.95							
ForvErs_1y		0.94	0.22						
ForvErs		0.94							
LoneInk_2y		0.92	0.25						
ForvErs_2y		0.91	0.27						
LoneInk_1y_D		0.60	0.58					-0.37	
LoneInk_D		0.59	0.56					-0.37	
LoneInk_2y_D		0.44	0.62					-0.38	
DispInk04		0.31	0.37						
DispInk04_1y		0.25	0.34						
DispInk_D		0.25	0.52						
DispInkFam04_1y		0.21	0.31						
SumAldP03		-0.30			-0.44			0.27	
SumAldP03_2y		-0.31			-0.43			0.29	
SumAldP03_1y		-0.32			-0.45			0.29	
SumTjP_1y			0.87					0.31	
SumTjP			0.85					0.29	
SumTjP_2y			0.84					0.33	
SumTjp_D			0.58					0.70	
SumTjp_1y_D			0.49					0.78	
SumTjp_2y_D			0.39					0.78	
AldPens_D			0.34		-0.20	0.38		0.75	
DispInk_2y_D			0.32						
DispInkFam_D			0.30						
DispInk_1y_D			0.30						
PrivPens			0.29			0.59			
DispInkFam_2y_D			0.26						
DispInkFam04_2y			0.25						
DispInkFam_1y_D			0.25						
DispInk04_2y			0.25						
PrivPens_2y			0.24			0.92			
PrivPens_1y			0.24			0.91			
AldPens_1y_D			0.24			0.38		0.80	

DispInkFam04	0.23						
ForTid_1y_D		0.90					
ForTid_D		0.88					
SocInk_1y_D		0.85					
ForTid_2y_D		0.82					
SocInk_2y_D		0.81					
SocInk_D		0.71	0.28				
ForTid_2y		0.64					
ForTid_1y		0.61					
ForTid		0.57					
SocInk_2y		0.52	0.40				
SocInk_1y		0.44	0.63				
SocInk		0.29	0.88				
SjukRe_2y_D		0.26					
AldPens_2y_D		-0.20		0.38		0.79	
ArbErs			0.96				
SjukRe			0.96				
SjukRe_1y			0.74				
SjukRe_2y			0.49				
SjukRe_D			0.45				
nn			0.21		0.42		
PrivPens_1y_D				0.94			
PrivPens_D				0.94			
PrivPens_2y_D				0.94			
bft_12m_tot					0.92		
bft_12m_tot_c619					0.61		
bft_3m					0.60		
bft_4m					0.59		
bft_5m					0.58		
bft_2m					0.57		
q4_t					0.53		
bft_1m					0.52		
q3_t					0.44		
c_tot_bw					0.35		
msv_days_c619					0.34		
q2_t					0.34		
msv_tot_prop					0.33		
bfd_12m					0.33		
bfd_60m					0.33		
msv_days					0.33		
msv_tot_prop_c619					0.30		
h_bft_3m					0.25		
q1_t					0.24		
h_tot_bw					0.22		
InkFNetto_1y							0.97
InkFNetto_2y							0.96
InkFNetto							0.95
InkFNetto_1y_D							0.73
InkFNetto_2y_D							0.71
InkFNetto_D							0.70
