# Supplementary material for: Study protocol for a comparative effectiveness evaluation of abiraterone acetate against enzalutamide: a longitudinal study based on Swedish administrative registers
Source: BMJ Open. 2021 Oct 22;11(10):e052610. doi: 10.1136/bmjopen-2021-052610 (PMC8547362; doi:10.1136/bmjopen-2021-052610)
Supplement: Author's manuscript [file bmjopen-2021-052610.draft_revisions.pdf]

BMJ Open is committed to open peer review. As part of this commitment we make the peer review history of every article we publish publicly available.

When an article is published we post the peer reviewers' comments and the authors' responses online. We also post the versions of the paper that were used during peer review. These are the versions that the peer review comments apply to.

The versions of the paper that follow are the versions that were submitted during the peer review process. They are not the versions of record or the final published versions. They should not be cited or distributed as the published version of this manuscript.

BMJ Open is an open access journal and the full, final, typeset and author-corrected version of record of the manuscript is available on our site with no access controls, subscription charges or pay-per-view fees (http://bmjopen.bmj.com).

If you have any questions on BMJ Open's open peer review process please email info.bmjopen@bmj.com

BMJ Open

# A study protocol for a comparative effectiveness evaluation of Abiraterone Acetate against Enzalutamide: A longitudinal study based on Swedish administrative registers

| | |
|---|---|
| Journal: | *BMJ Open* |
| Manuscript ID | bmjopen-2021-052610 |
| Article Type: | Protocol |
| Date Submitted by the Author: | 21-Apr-2021 |
| Complete List of Authors: | Johansson, Per; Uppsala Universitet Humanistisk-samhallsvetenskapliga vetenskapsomradet, Statistics<br>Jonéus, Paulina; Uppsala University, Department of Statistics<br>Langenskiöld, Sophie; Uppsala University, Department of Medical Sciences; Uppsala University |
| Keywords: | Protocols & guidelines < HEALTH SERVICES ADMINISTRATION & MANAGEMENT, EPIDEMIOLOGY, Epidemiology < ONCOLOGY, Urological tumours < ONCOLOGY, PUBLIC HEALTH, Prostate disease < UROLOGY |
| | |

SCHOLARONE™
Manuscripts

*I, the Submitting Author has the right to grant and does grant on behalf of all authors of the Work (as defined in the below author licence), an exclusive licence and/or a non-exclusive licence for contributions from authors who are: i) UK Crown employees; ii) where BMJ has agreed a CC-BY licence shall apply, and/or iii) in accordance with the terms applicable for US Federal Government officers or employees acting as part of their official duties; on a worldwide, perpetual, irrevocable, royalty-free basis to BMJ Publishing Group Ltd ("BMJ") its licensees and where the relevant Journal is co-owned by BMJ to the co-owners of the Journal, to publish the Work in this journal and any other BMJ products and to exploit all rights, as set out in our licence.*

*The Submitting Author accepts and understands that any supply made under these terms is made by BMJ to the Submitting Author unless you are acting as an employee on behalf of your employer or a postgraduate student of an affiliated institution which is paying any applicable article publishing charge ("APC") for Open Access articles. Where the Submitting Author wishes to make the Work available on an Open Access basis (and intends to pay the relevant APC), the terms of reuse of such Open Access shall be governed by a Creative Commons licence – details of these licences and which Creative Commons licence will apply to this Work are set out in our licence referred to above.*

*Other than as permitted in any relevant BMJ Author's Self Archiving Policies, I confirm this Work has not been accepted for publication elsewhere, is not being considered for publication elsewhere and does not duplicate material already published. I confirm all authors consent to publication of this Work and authorise the granting of this licence.*

For peer review only

# A study protocol for a comparative effectiveness evaluation of Abiraterone Acetate against Enzalutamide: A longitudinal study based on Swedish administrative registers

Per Johansson, PhD[1]; Paulina Jonéus[1]; Sophie Langenskiöld, PhD[2]

1. Department of Statistics, Uppsala University, Sweden
2. Department of Medical Sciences, Uppsala University, Sweden

Address for correspondence

Sophie Langenskiöld, PhD

Department of Medical Sciences, Uppsala University, Sweden

E-mail: Sophie.langenskiold@medsci.uu.se; Mobile: +46(0)709 393733

For peer review only

**Abstract**

**Introduction**: This paper presents a study protocol for a comparative effectiveness evaluation of abiraterone acetate against enzalutamide, two cancer drugs, used with patients suffering from advanced prostate cancer.

**Method and analysis**: The study protocol uses a matching design  on pre-treatment covariates. A two-step procedure is recommended in choosing relevant covariates in the matching design when there is a substantial number of covariates. First, an exploratory factor analysis reduces the dimension of a large set of continuous covariates to nine factors. In addition, we reduce the dimension of the covariates, interactions and second order terms for the continuous covariates using propensity score estimation. Second, the final design makes use of a genetic matching algorithm. **Results:** While the matching design yields a balance on the observed covariates, one cannot discard the possibility that unobserved confounders are not balanced. For that reason, a sensitivity test for this assumption is also presented **Ethics and dissemination:** The study was approved by the Regional Ethical Review Board in Uppsala, Sweden (Dnr 2017/482).

**Keywords:** protocol, comparative effectiveness research, real world evidence, metastatic castration-resistant prostate cancer, abiraterone acetate, enzalutamide, mortality, morbidity

For peer review only

**Strengths and limitations of this study:**

- The study uses up-to-date mixed methods to design an observational study for a comparative effectiveness study of abiraterone acetate against enzalutamide on four outcomes.
- The study replicates a randomised study ex-post, by achieving comparable groups on patients' historical health and socioeconomic status, based on rich, linked, population registries.
- While the quasi-experimental design yields a balance on observed covariates, unobserved covariates may still differ across the groups. Sensitivity analyses to evaluate potential remaining confounding are thus suggested in the protocol.
- Mortality data and data to test for remaining confounding will be added after this publication. The design is consequently not affected by the main outcome, and the test will be validated.

For peer review only

# Introduction

Prostate cancer (PC) is the most commonly diagnosed form of cancer in Sweden. In 2016, for example, 10,473 additional patients were diagnosed with prostate cancer, creating a pool of 107,752 patients.[1,2] PC is also the second leading cause of cancer death, and almost all mortalities arise when the patients have progressed to the advanced stage, metastatic castrate resistant prostate cancer (mCRPC).

Various treatment alternatives are available for patients with mCRPC. In the last two decades, chemotherapy and novel antiandrogenic (NAM) medications have revolutionised the treatment in mCRPC patients.[3-8] This paper presents a study protocol for the design of a comparative effectiveness evaluation of two of these NAMs, i.e. abiraterone acetate (AA) in combination with prednisone and Enzalutamide (ENZ). The evaluation concerns their use in clinical practice from June 2015, which corresponds to the period when mCRPC patients were reimbursed for these drugs.

Data are collected from population registers, administrated by the National Board of Health and Welfare (NBHW) and Statistics Sweden (SCB). The population is restricted to all men in the NBHW register with a prostate cancer diagnosis before 2017, as only these patients were expected to progress to mCRPC during the period for which we had outcome data. We restrict the population to patients prescribed AA or ENZ during the period 1 June 2015 to 15 June 2018, i.e. the period during which AA and ENZ were only prescribed for mCRPC.

The aim of the design is to estimate the comparative effectiveness of AA against ENZ. This will be done using detailed data from the population registers and up to date statistical methods, combined with an understanding of the patients' prescription patterns. Such information will be obtained from a qualitative study with twelve urologists and oncologists, with different specialisations at different hospitals. The main outcome of interest is overall mortality. Mortality data will be added after the publication of this study design. In addition,

For peer review only

the comparative effectiveness on two secondary outcomes (pain and skeleton related events) will be studied. Treatment length and prostate specific mortality will be added as exploratory endpoints.

# Method and analysis

### Study design and research questions

A nationwide registry-based longitudinal study using patient-level data from multiple population registries administrated by NBHW and SCB. Data from these registries are linked using a unique serial number created by SCB.

*Study population*

The population under study is defined using the cancer registry. We began by identifying all men with a prostate cancer diagnosis before 2017, as well as the year of their diagnosis. Thereafter, we identified 243,535 unique patients with prostate cancer (ICD10 code C61.9 or earlier codes ICD7 177 and ICD9 185.9). If a patient, for some reason, has been diagnosed multiple times with this diagnosis, the first observed time period is used (1,661 patients appear multiple times; 1,623 of these diagnosed on two or more different dates; and 181 patients are diagnosed at different hospitals).

The fraction (the number of diagnoses divided by the number of men in the population) of diagnoses per year in our data from 1986 to 2016 is presented in Figure S1 in the supplementary material. The figure shows more than 100% increase in the prevalence over the years; from just above 0.1% in 1986 to more than 0.2% in 2016.

The population is restricted to all men collecting a prescription of AA or ENZ during the period 1 June 2015 to 15 June 2018. The reasons for the time restriction are: (i) that almost no one was treated with these drugs before the reimbursement of AA and ENZ in June and July 2015, respectively and (ii) that AA was additionally reimbursed in combination with

For peer review only

ADT, in patients with high-risk castration sensitive prostate cancer (mHSPC) and unsuited for docetaxel in 15 June 2018.

The restriction leaves us with a total of 4,601 patients in the study population. Some of the patients were prescribed AA or ENZ before the subsidisation but collected the drug after June 2015; these individuals are included in the sample. For this population, the year of the diagnosis ranges between 1986 and 2016. Consequently, there is substantial variation in the time when prescribed AA or ENZ and the date of diagnosis. This so-called time to prescription is most likely an important covariate.

About 10% of the patients were prescribed both AA and ENZ over the years. We allocate these patients to the two samples, AA and ENZ-takers, based on their first prescription of one of the two drugs. Thus, the results should be interpreted as an intention to treat analysis. The number of AA and ENZ patients in the study population each year is presented in Table S1 in the supplementary material. We can see that the number of treated patients continues to increase until 2016. The reason for the drop in 2018 is that we only have data until 15 June 2018.

The prescription for the two drugs varies over the 21 county councils, hereinafter counties, the responsible body for health care in Sweden. The proportions of AA and ENZ for the periods 2015 to 2018 in the 21 counties are presented in Table 1. The fact that the prescription varies over counties is a notable finding, as it suggests differences in prescription that may, for instance, be driven by practice variation. From this table, we can also see that across the counties, on average, 24% of the patients were prescribed AA.

Table 1: Proportion prescribed enzalutamide and abiraterone respectively, per county and in total

For peer review only

| | Enzalutamide | | | | | Abiraterone | | | | |
|---|---|---|---|---|---|---|---|---|---|---|
| County | 2015 | 2016 | 2017 | 2018 | TOT | 2015 | 2016 | 2017 | 2018 | TOT |
| Blekinge | 0.84 | 0.91 | 0.84 | 0.80 | 0.85 | 0.16 | 0.09 | 0.16 | 0.20 | 0.15 |
| Dalarna | 0.86 | 0.65 | 0.55 | 0.62 | 0.69 | 0.14 | 0.35 | 0.45 | 0.38 | 0.31 |
| Gavleborg | 0.60 | 0.92 | 0.93 | 0.96 | 0.85 | 0.40 | 0.08 | 0.07 | 0.04 | 0.15 |
| Gotland | 0.75 | 0.92 | 1.00 | 1.00 | 0.89 | 0.25 | 0.08 | | | 0.11 |
| Halland | 0.75 | 0.89 | 0.88 | 0.76 | 0.82 | 0.25 | 0.11 | 0.12 | 0.24 | 0.18 |
| Jamtland | 0.47 | 0.81 | 0.77 | 0.67 | 0.70 | 0.53 | 0.19 | 0.23 | 0.33 | 0.30 |
| Jonkopings lan | 0.62 | 0.91 | 0.89 | 0.76 | 0.80 | 0.38 | 0.09 | 0.11 | 0.24 | 0.20 |
| Kalmar | 0.74 | 0.89 | 0.83 | 0.93 | 0.84 | 0.26 | 0.11 | 0.17 | 0.07 | 0.16 |
| Kronoberg | 0.48 | 0.43 | 0.21 | 0.20 | 0.37 | 0.52 | 0.57 | 0.79 | 0.80 | 0.63 |
| Norrbotten | 0.83 | 0.88 | 0.64 | 0.65 | 0.74 | 0.17 | 0.12 | 0.36 | 0.35 | 0.26 |
| Orebro | 0.77 | 0.95 | 0.68 | 0.38 | 0.74 | 0.23 | 0.05 | 0.32 | 0.62 | 0.26 |
| Ostergotlands lan | 0.12 | 0.90 | 0.88 | 0.90 | 0.83 | 0.88 | 0.10 | 0.12 | 0.10 | 0.17 |
| Skane | 0.87 | 0.96 | 0.96 | 0.85 | 0.92 | 0.13 | 0.04 | 0.04 | 0.15 | 0.08 |
| Sodermanland | 0.77 | 0.98 | 1.00 | 0.92 | 0.91 | 0.23 | 0.02 | | 0.08 | 0.09 |
| Stockholm | 0.71 | 0.82 | 0.80 | 0.79 | 0.78 | 0.29 | 0.18 | 0.20 | 0.21 | 0.22 |
| Uppsala | 0.62 | 0.56 | 0.25 | 0.36 | 0.46 | 0.38 | 0.44 | 0.75 | 0.64 | 0.54 |
| Varmland | 0.87 | 0.96 | 0.67 | 0.75 | 0.83 | 0.13 | 0.04 | 0.33 | 0.25 | 0.17 |
| Vasterbotten | 0.63 | 0.70 | 0.62 | 0.76 | 0.67 | 0.37 | 0.30 | 0.38 | 0.24 | 0.33 |
| Vasternorrland | 0.47 | 0.71 | 0.73 | 0.86 | 0.64 | 0.53 | 0.29 | 0.27 | 0.14 | 0.36 |
| Vastmanland | 0.73 | 0.79 | 0.80 | 0.55 | 0.74 | 0.27 | 0.21 | 0.20 | 0.45 | 0.26 |
| Vastra gotalands lan | 0.51 | 0.78 | 0.69 | 0.65 | 0.66 | 0.49 | 0.22 | 0.31 | 0.35 | 0.34 |
| Total | 0.68 | 0.83 | 0.76 | 0.74 | 0.76 | 0.32 | 0.17 | 0.24 | 0.26 | 0.24 |

*The Data source*

From the NBHW, we link data from the inpatient care register and the pharmaceutical register. All in- and out-patient care visits in Sweden and all prescribed drugs are registered in these registers. The in-patient care register contains, among other things, information on all diagnoses (using the ICD classifications), the date of admission, and discharge. The pharmaceutical register contains the date when the drug was prescribed and dispensed, as well as the ATC class of the drug.

From SCB, we link data from a census conducted every fifth year during the period 1960–2015, as well as labour statistics based on administrative sources (RAMS) or LISA for the period 1985–2015. RAMS and LISA are large databases, created by linking a large set of

For peer review only

administrative registers using the Swedish personal identification number in the linkage. The linked data contains disposable income, labour income, social insurance payments, capital income, labour market status, year of birth, education, marital status, etc. for each individual for the period 1960–2015.

*Confounding*

Groups participating in the different interventions can only be compared if they are identical on all those variables which simultaneously influence their prescriptions and outcomes. Therefore, we first conducted a qualitative study, with the primary focus to understand who prescribed which of the drugs, when, and for how long. This study is soon to be submitted for publication (Sophie Langenskiöld 2021).

The responses from the interviews were heterogeneous. There is however quite good agreement on the following three statements that are relevant for this paper. Firstly, ENZ is preferred for patients with cardiovascular diseases, diabetes, or osteoporosis, whereas AA is preferred for patients with poor general health condition and fatigue. Secondly, doctors want their patients to benefit from as many treatment options as possible during their lifetime. If it turns out that patients' general health is good and the disease is progressing rapidly, chemotherapy is the preferred treatment. On the other hand, if it turns out that patients' general health is poor and the disease is progressing slowly, then ENZ or AA is the preferred treatment. Thirdly, doctors monitor the treatment and if it is found that treatment is ineffective or intolerable, it is stopped.

*Variables/Covariates*

From the in-patient care registers and the pharmaceutical register, we have extensive historical information on patients' health and health care consumption. This enables us to create an almost infinite number of covariates. We use health data for the period up to five

For peer review only

years before the diagnosis and exclude health data from the date of treatment and onward. Hence, all covariates are measured before the first prescription of AA or ENZ and thus cannot be affected by treatment.

To exemplify the available data, the medical history from the in-patient care and prescription registry of a randomly chosen patient is presented in Figure 1. This patient was diagnosed with prostate cancer in October 2007 (i.e. a C61.9 ICD-10 code) and was first treated with ENZ in April 2018 (i.e. a L02BB04 ATC code). This patient had no in-patient care visits the years prior to the cancer diagnosis, and the visits after the diagnosis are mainly related to the prostate cancer diagnosis. The patient was hospitalised for more than one consecutive day on two occasions.

[INSERT FIGURE 1 HERE]

Figure 1: One randomly chosen enzalutamide-taker

The medical history data are aggregated separately over a five-year period before the diagnosis and for the period in-between diagnosis and prescription. From the data, we derive, among other things, the number and length of health care visits over the two periods. This is also done separately for metastases, cardiovascular, secondary, diabetes, and fatigue or osteoporosis diagnosis. We also derive information about the number of specific drugs collected between the cancer diagnosis and treatment, separately for prescriptions related to these diagnoses.

To gather information on sickness progression, we separately calculated the number of visits during the last months before treatment and calculated the average number of visits per quarter between diagnosis and treatment.

Using this strategy, we created 45 continuous covariates, where 13 measure the health status before the prostate cancer diagnosis. We also identified 22 indicator variables, of which

For peer review only

seven measure health status before diagnosis, including whether a patient has had a diagnosis related to a metastasis, cardiovascular disease, diabetes, fatigue or osteoporosis. See Table S2 in the supplementary material for the included ICD codes.

From the SCB data, we created 94 variables that supposedly will be able to describe the socioeconomic status of the patient, both three years before the diagnosis and the treatment, with data from 1991 until 2015. For patients with a diagnosis before or after these years, information on socioeconomic status is given by values from a year as close to the diagnosis as possible. This includes information on age, marital status and educational level as well as pensions, income, sick leave and other security benefits for the patient and the household. For any missing values, the mean over the three preceding years is used. Educational level is the highest completed education and is classified as less than, equal to, or more than secondary school.

Besides the 161 covariates, we also added historical county specific mortality for prostate cancer. That is, the mean number of men per 100,000 inhabitants who died from prostate cancer during the period 1997–2017. All 162 covariates, including descriptions, are presented in Table S3 in the supplementary material. Table 2 provides summary statistics of the AA and ENZ patients for the subset of covariates deemed to be the most important. From this table, we can see that the two groups in general are very similar; there are, for instance, no significant differences in average age, educational level or marital status.

Table 2: Summary statistics

| **Description** | **ENZ** | **AA** | **Diff** |
|---|---|---|---|
| Age at treatment | 75.27 (7.85) | 75.29 (7.70) | -0.02 |
| Years to treatment from diagnosis | 6.95 (5.00) | 7.29 (5.31) | 0.34* |
| Less than secondary school education | 0.36 (0.48) | 0.35 (0.48) | 0.01 |
| Secondary school education | 0.39 (0.49) | 0.39 (0.49) | -0.00 |

For peer review only

| | | | |
|---|---|---|---|
| More than secondary school education | 0.25 (0.43) | 0.26 (0.44) | -0.01 |
| Living with a partner | 0.66 (0.47) | 0.67 (0.47) | -0.01 |
| Cardiovascular disease before treatment | 0.35 (0.48) | 0.34 (0.47) | 0.01 |
| Atrial fibrillation and flutter before treatment (I48) | 0.16 (0.36) | 0.18 (0.38) | -0.02 |
| Acute myocardial infarction before treatment (I21) | 0.10 (0.30) | 0.07 (0.25) | 0.03*** |
| Other cardiovascular diseases before treatment | 0.27 (0.45) | 0.26 (0.44) | 0.01 |
| Diabetes before treatment | 0.16 (0.37) | 0.12 (0.32) | 0.04*** |
| Osteoporosis before treatment | 0.01 (0.11) | 0.01 (0.10) | 0.00 |
| Secondary malignant neoplasms (metastases) | 0.70 (0.46) | 0.75 (0.43) | 0.05*** |
| Malaise and fatigue | 0.05 (0.22) | 0.04 (0.20) | 0.01 |

*Standard deviations within parentheses. * p <0.1; ** p <0.05; *** p <0.01.*

**Statistical analysis plan**

*The matching*

The genetic matching approach of Diamond and Sekhon is used in the design.[9] This is a generalisation of Mahalanobis' distance matching, where an evolutionary search algorithm developed by Sekhon and Mebane is used to maximise the balance of a set of observed covariates together with an estimated propensity score.[10]

Due to computational limitations, we cannot match on all covariates. Thus, we need to restrict the set to those deemed the most important. According to the twelve urologists and oncologists interviewed for this project, cardiovascular diseases, fatigue, diabetes, osteoporosis and age at treatment influenced their choice of prescriptions and were thus included. As prevalence of visceral metastases indicated the severity of the disease, and the sequencing of prostate-cancer drugs reflected the optimisation of care according to the same specialists, we also considered presence of visceral metastases and time to prescription. Also, socioeconomic variables such as educational level, marital status and quality of care differences across counties, such as those reflected in the county specific mortality, were



For peer review only

included. We also include nine factors that summarise the information from the 130 remaining covariates, see Table S3, and an estimated propensity score.

The factors are derived from an exploratory factor analysis (using the varimax rotation and factor scores derived using the regression method.[11,12] The nine factors account for 42 per cent of the variance in the 130 variables. See Table S4 and Table S5 in the supplementary material for the factor analysis results and a list of the interpretation of the factors.

The propensity score is estimated using the logit model and LASSO regression.[13] All 162 covariates are included; in addition, interactions and second order polynomials of the continuous covariates are included. The LASSO regression has a penalty, or cost, of including too many covariates. This helps us to avoid the risk of over-parametrising the propensity-score, which may lead to bias.[14] The results from the finally estimated propensity score model is presented in Table S6 in the supplementary material.

A one-to-one matching with replacement is used in the genetic matching approach, but we exclude observations with a distance that is more than three standard deviations for any of the included covariates. This leads to 85 dropped observations and a total of 4,516 matched observations.

The propensity score balance is presented in Figure 2. It is clear that the estimated probability of being treated is similar already in the unmatched groups, but the balance is improved in the adjusted sample.

[INSERT FIGURE 2 HERE]

Figure 2: Propensity score overlap, before and after matching

The covariate balance in the matched sample is examined through the standardised difference in per cent of the average standard deviation (SB). Further, we examine the variance ratio

For peer review only

(VR) between the two groups for each of the included covariates. In a well conducted randomised experiment, we expect the SB to be no larger than 25 per cent and the VR to be close to one. This is used as a rule of thumb in the quasi-experimental literature.[15] The results are presented in Table 3.

Table 3: Standardized difference in percent and variance ratio before and after matching

| | SB before | SB after | VR before | VR after |
|---|---|---|---|---|
| Age (Years) | 0.28 | 1.10 | 0.96 | 0.90 |
| Diabetes (Y/N) | 12.48 | 0.70 | 0.77 | 0.99 |
| Other CVD (Y/N) | 3.65 | 1.77 | 0.96 | 0.98 |
| Acute myocardial infarction, I21 (Y/N) | 10.99 | 0.00 | 0.72 | 1.00 |
| Atrial fibrillation and flutter, I48 (Y/N) | 5.43 | 0.91 | 1.10 | 0.98 |
| Osteoporosis (Y/N) | 1.79 | 0.00 | 0.85 | 1.00 |
| Fatigue (Y/N) | 4.56 | 0.00 | 0.82 | 1.00 |
| Less than secondary school education | 3.15 | 5.08 | 0.98 | 1.03 |
| Secondary school education | 0.45 | 2.32 | 1.00 | 0.99 |
| Time to treatment (Years) | 6.58 | 0.55 | 1.13 | 0.94 |
| Secondary malignant neoplasms (Y/N) | 11.73 | 1.48 | 0.89 | 0.99 |
| Time to treatment squared (Years) | 7.75 | 1.26 | 1.23 | 0.88 |
| Age squared (Years) | 0.07 | 0.58 | 0.97 | 0.91 |
| Age and Diabetes | 12.41 | 0.45 | 0.77 | 1.00 |
| Age and CVD | 3.44 | 1.43 | 0.97 | 0.99 |
| Age and Secondary malignant neoplasms | 12.06 | 1.59 | 0.90 | 0.98 |
| Diabetes and CVD | 6.77 | 0.00 | 0.77 | 1.00 |
| County specific mortality | 6.05 | 0.23 | 0.99 | 0.90 |
| Partner (Y/N) | 4.01 | 6.33 | 0.94 | 0.90 |
| Factor 1 | 1.03 | 1.48 | 1.08 | 0.72 |
| Factor 2 | 1.29 | 0.54 | 0.36 | 0.94 |
| Factor 3 | 0.66 | 5.03 | 0.68 | 0.81 |
| Factor 4 | 5.07 | 2.39 | 1.42 | 0.94 |
| Factor 5 | 4.37 | 1.48 | 0.71 | 0.95 |
| Factor 6 | 5.54 | 3.35 | 0.13 | 0.76 |
| Factor 7 | 1.30 | 1.90 | 0.53 | 0.78 |
| Factor 8 | 0.15 | 0.43 | 0.90 | 0.90 |
| Factor 9 | 0.21 | 0.35 | 0.48 | 0.97 |

For peer review only

| | | | | |
|---|---|---|---|---|
| Propensity score | 37.03 | 9.10 | 0.76 | 0.83 |

**Planned analysis**

We have one primary and two secondary outcomes. The primary outcome is all-cause mortality (DEAD), and the two secondary outcomes capturing morbidity are PAIN and SRE. PAIN is an indicator for severe pain, and SRE is an indicator for a skeleton-related event.

The reason for including PAIN and SRE as outcomes is that these two morbidities have been seen as common complications of bone metastases.[16,17] The reason for studying COMPLY as an exploratory outcome is that it captures how the treatments can emain effective and/or safe. According to our qualitative work, the main reasons for treatment interruptions is a lack of safety and being effective.

For the inference, we will use Bonferroni correction with a five per cent overall level. With one primary and two secondary outcomes, this means that the significance level on the single outcomes will be 1.67% (= 100*0.05/3).

DEAD as a primary endpoint is an indicator variable, defined as one for patients who died due to any causes and zero for other patients at the end of each 30-day period after being prescribed AA or ENZ. Mortality data will be available until the end of June 2020. Consequently, the first patient administered the treatments can theoretically have up to 70 mortality registrations, but the actual number of registrations will most likely be fewer as they suffer from a deadly disease.

Patients are assumed to suffer severe pain if they receive prescriptions for neuropathic pain, i.e. opiates in combination with tramadol and paracetamol (ATC-codes N02AA, N02AX02,

For peer review only

and N02BE01). The PAIN indicator is one for periods in which the patient has received such a subscription and zero for the other periods. Prescription data will be available until 31st of December 2020. Therefore, up to 76 periods are available for prescription data.

Patients are assumed to suffer skeleton-related event if they are hospitalised because of pathologic fracture (ATC codes M485, M495, M844, and M907) or spinal cord compression (G550, G834, G952, G958, G959, and G992) (Parry et al., 2019). The SRE indicator is one for periods with such hospitalisations and zero for the other periods. As in-patient care data are available until 31st of December 2019, at most 64 periods are available.

For each of the periods, we will estimate the above three effects. We will display the Bonferroni adjusted confidence intervals (i.e. using the 1.67% level for an overall 5% level test) at each time period.

For all outcomes, regression analysis will be used in the estimation of the comparative effectiveness analyses of AA against ENZ. We adjust for all variables displayed in Table 3, except for the propensity score. In addition, we allow for heterogeneous effects by also adjusting for the covariates interacted with AA. For the inference, we will use the Eicker-Huber-White (EHW) covariance estimator robust covariance matrix.[18-20]

The problem with analysis of the two morbidity outcomes is that they are only observed in the data if the patient is alive. In the analysis above, at each evaluation period, we will exclude the dead patients from the analysis. This means that the number of valid observations will be reduced over the evaluation window. If there are no differences in mortality rates between the two drugs, this analysis strategy provides unbiased estimates of the comparative effectiveness on morbidity. However, if there are differences in mortality rates, these analyses are biased, as it is more likely that we will observe a morbidity for the drug with lower mortality rates. If this is the case, we will need to estimate bounds of potential effects

For peer review only

as a sensitivity analysis. We labelled all patients who died as either having the morbidity or not (i.e. PAIN =1 and SRE=1 and PAIN =0 and SRE=0). If the mortality is observed to be higher than for ENZ, the first case provides the upper bound estimate of the comparative effectiveness of AA against ENZ while the second one provides the lower bound on these two morbidity outcomes, and vice versa if the mortality rate is lower for AA.

In addition, we will analyse two explanatory endpoints. The first is prostate-specific mortality, and the second is the Duration of Reversing Treatments (DRT). Prostate-specific mortality is measured in 30-day intervals in the same way as in the main analysis. DRT is measured as the time until the patient is observed to have stopped the treatment. DRT is calculated based on prescription data and their average estimated compliance rate.

The problem with analysing prostate-specific mortality relates to how to deal with people dying from other causes of death. To this end, we repeat the analysis with the two morbidity outcomes, that is, we exclude patients who die from other causes and also estimate the upper and lower bound where those who die from other causes are either treated as dying from prostate cancer or not dying. All estimates are performed using the regression models explained above. The analysis of DRT will be conducted using Cox regression. Covariates displayed in Table 3 will be added in addition to the indicator for AA. We treat mortality as a competing risk, conditional on the observed covariates.

**Sub-analyses**

Sub-analyses on these four outcomes will be conducted on patients who are believed (i) to be potential high-/low responders, (ii) suffer a more aggressive disease, and (iii) have a long or short time until prescription.

Using the results from our qualitative work, we define high responder patients (i) as those who are treated twelve months or more with a previous hormone treatment, i.e. LHRH

For peer review only

agonists or –antagonists, while the low responder is treated for a shorter period. The hormone treatments are Leuprolide (ATC L02AE02), Goserelin (ATC L02AE03), Triptorelin (ATC L02AE04), Histrelin (ATC L02AE05) and Degarelix (ATC L02BX02), respectively. This corresponds to 63 per cent of the sample being categorised as high respondents.

We define patients with visceral metastases in their hospital records (ICD-10 code C78) before they start their AA or ENZ treatment as having have more aggressive disease (ii).[21,22] About 7 per cent of the patients are defined as suffering from a more aggressive disease.

All patients having a shorter period of time between diagnosis of prostate cancer and prescription for AA or ENZ treatment, as opposed to the median waiting time for both drugs are defined as patients with short time until subscription, (iii) while the complement is patients with long time until subscription.

## Public and private involvement

Clinicians (Oncologists and Urologist) were involved in previous works for this protocol development. The clinicians were interviewed to understand factors that are important while prescribing AA and ENZ.

## Ethical approval

Ethical approval was granted by the Ethical committee in Uppsala (ref. Dnr2017/482).

## Discussion

The aim of this paper was to present a study protocol for the evaluation of the comparative effect of AA against ENZ for prostate cancer patients. The study design is based on the quasi-experimental Rubin Causal Model and makes use of matching on pre-treatment covariates.[23] Due to the substantial number of covariates, a two-step procedure is recommended for

For peer review only

choosing relevant covariates in the matching design. In the first step, a factor analysis reduces the dimension of a large set of continuous covariates to nine factors. In the second step, we reduce the dimension of the covariates, and second order terms for the continuous covariates, using propensity score estimation. The final design makes use of a genetic matching algorithm, using, in total, 26 covariates together with the propensity score.

While the matching design yields balanced observed covariates, this is an observational study with the usual limitations in this context. In particular, one cannot discard the possibility that unobserved confounders are not balanced. However, Rosenbaum's sensitivity analysis for hidden bias will be used.[24] McNemar's test will be used when calculating ranges of p-values of the Intent to treat effect for different values of the sensitivity parameter.[25]

In addition, we will add data from the national prostate cancer register (NPCR). These data contain more detailed information on patient's heath with regard to the prostate cancer but do not have full coverage. We have requested the data and will have access to it at the same time as when we have the mortality data. These data allow us to basically test the validity of the design by estimating the effects on potential pre-treatment confounders. If there is a statically significant effect on these covariates, this suggests that available data from the population register are not sufficient to control for confounding bias.

For the test, we use three covariates, which during discussions with specialists, are judged to be important confounders: PSA levels (D_SPSA), Gleason score (D_GleasSa), and metastases (D_Mstad) at the time of the prostate cancer diagnosis. With three pre-measured covariates, as in the main analysis, we adjust the significance level for the individual tests using Bonferroni correction based on a five per cent overall level. This means that the level for testing on each single outcome is 1.67 per cent. For all three covariates, regression analysis will be used in the estimation, using the same regression designs as used for the main analysis. NPCR also

For peer review only

allows us to conduct more exploratory subgroup analyses. Since one of the drug companies we consulted believed that the initial treatment may influence the effectiveness of their drug, we will separately evaluate patients who receive conservative therapy (wait and see), radical prostatectomy, and radiation therapy as their primary treatment.

Generalisability of the results to other populations may not necessarily be granted, for instance, if future treated populations differ greatly in characteristics, which modifies the effect of the treatment.

For peer review only

**Author contributions**

PJ and SL conceived the main conceptual ideas to replicate a randomised study ex-post using Swedish population registries. SL lead and planned the project, and carried out the preparatory qualitative work. PJ, together with PaJ, worked out the technical details in discussion with SL, and performed the analytical computations. All authors discussed the results and contributed to the final manuscript.

**Funding statement**

This work was supported by the Dental and Benefit Agency [diary number 02823/2017]

**Competing interest**

The authors declare that they have no competing interests.

**Patient consent for publication**

Not applicable.

**Acknowledgements**

We would like to acknowledge the valuable contributions of the urologists and oncologists who participated in our qualitative work (R. Blom, G. Ahlgren, Å. Jellvert, M. Anden, K. Holmsten, S-O. Andersson, L. Nygren, L. Åström, M. Hellström, C. Thellenberg, J. Yachnin, J. Sandzen). They helped us understand what factors govern the prescription of AA and ENZ, i.e. factors which need to be balanced in any groups compared in a comparative effectiveness evaluation. Also, we would like to thank the groups of experts with whom we interacted in order to improve our protocol, i.e. either the balance across the groups or the endpoints to focus on (A. Bill-Azelsson, D. Robinson, R. Henriksson, and representatives from Astellas Pharma and Janssen Inc., i.e. Kerstin Magnusson, Johanna Svensson, Ingrid Tredal, and Julia Rockberg, respectively. Finally, we thank Sven-Åke Lööf for sharing his great understanding of the field.

# References

1. Fitzmaurice C, Abate D, Abbasi N, *et al.* Global, regional, and national cancer incidence, mortality, years of life lost, years lived with disability, and disability-adjusted life-years for 29 cancer groups, 1990 to 2017: a systematic analysis for the global burden of disease study. *JAMA Oncol* 2019;**5**(12):1749–1768.
2. Socialstyrelsen. Cancer i siffror 2018 populärvetenskapliga fakta om cancer, Cancerfonden och Socialstyrelsen i samarbete. https://www.socialstyrelsen.se/globalassets/sharepoint-dokument/artikelkatalog/statistik/2018-6-10.pdf Published 2018. Accessed 15 October 2019.
3. Basch E, Autio K, Ryan CJ, *et al.* Abiraterone acetate plus prednisone versus prednisone alone in chemotherapy-naive men with metastatic castration-resistant prostate cancer: patient-reported outcome results of a randomised phase 3 trial. *Lancet Oncol* 2013;**14**(12):1193–1199.
4. Beer TM, Armstrong AJ, Rathkopf DE, *et al.* Enzalutamide in metastatic prostate cancer before chemotherapy. *N Eng J Med* 2014;**371**(5):424–433.

For peer review only

5. Loriot Y, Miller K, Sternberg CN, *et al.* Effect of enzalutamide on health-related quality of life, pain, and skeletal-related events in asymptomatic and minimally symptomatic, chemotherapy-naive patients with metastatic castration-resistant prostate cancer (PREVAIL): results from a randomised, phase 3 trial. *Lancet Oncol* 2015;**16**(5):509–521.

6. Ryan CJ, Smith MR, De Bono JS, *et al.* Abiraterone in metastatic prostate cancer without previous chemotherapy. *N Eng J Med* 2013;**368**(2):138–148.

7. Tannock IF, De Wit R, Berry WR, *et al.* Docetaxel plus prednisone or mitoxantrone plus prednisone for advanced prostate cancer. *N Eng J Med* 2004;**351**(15):1502–1512.

8. Ye D, Huang Y, Zhou F, *et al.* A phase 3, double-blind, randomized placebo-controlled efficacy and safety study of abiraterone acetate in chemotherapy-naïve patients with mCRPC in China, Malaysia, Thailand and Russia. *Asian J Urol* 2017;**4**(2):75–85.

9. Diamond A, Sekhon JS. Genetic matching for estimating causal effects: A general multivariate matching method for achieving balance in observational studies. *Rev Econ Stat* 2013;**95**(3):932–945.

10. Sekhon JS, Mebane Jr WR. Genetic optimization using derivatives. *Political Anal* 1998:187–210.

11. Costello AB, Osborne J. Best practices in exploratory factor analysis: Four recommendations for getting the most from your analysis. *Pract Assess Res Evaluation* 2005;**10**(1):7.

12. DiStefano C, Zhu M, Mindrila D. Understanding and using factor scores: Considerations for the applied researcher. *Pract Assess Res Evaluation* 2009;**14**(1):20.

13. Tibshirani R. Regression shrinkage and selection via the lasso. *J R Stat Soc Series B Methodol* 1996;**58**(1):267–288.

14. De Luna X, Waernbaum I, Richardson TS. Covariate selection for the nonparametric estimation of an average treatment effect. *Biometrika.* 2011;**98**(4):861–875.

15. Imbens GW, Rubin DB. *Causal inference in statistics, social, and biomedical sciences.* Cambridge University Press; 2015.

For peer review only

16. Drudge-Coates L, Oh WK, Tombal B, *et al.* Recognizing symptom burden in advanced prostate cancer: a global patient and caregiver survey. *Clin Genitourin Cancer* 2018;**16**(2):e411–e419.

17. So A, Chin J, Fleshner N, Saad F. Management of skeletal-related events in patients with advanced prostate cancer and bone metastases: Incorporating new agents into clinical practice. *Can Urol Assoc J* 2012;**6**(6):465.

18. Eicker F. Limit theorems for regressions with unequal and dependent errors. Paper presented at: Proceedings of the fifth Berkeley symposium on mathematical statistics and probability 1967.

19. Huber PJ. The behavior of maximum likelihood estimates under nonstandard conditions. Paper presented at: Proceedings of the fifth Berkeley symposium on mathematical statistics and probability 1967.

20. White H. A heteroskedasticity-consistent covariance matrix estimator and a direct test for heteroskedasticity. *Econometrica: journal of the Econometric Society* 1980:817–838.

21. Mazzone E, Preisser F, Nazzani S, *et al.* Location of metastases in contemporary prostate cancer patients affects cancer-specific mortality. *Clin Genitourin Cancer* 2018;**16**(5):376–384. e371.

22. Whitney CA, Howard LE, Posadas EM, *et al.* In Men with Castration-Resistant Prostate Cancer, Visceral Metastases Predict Shorter Overall Survival: What Predicts Visceral Metastases? Results from the SEARCH Database. *Eur Urol Focus* 2017;**3**(4-5):480–486.

23. Rubin DB. Estimating causal effects of treatments in randomized and nonrandomized studies. *J Educ Psychol* 1974;**66**(5):688.

24. Rosenbaum PR. Overt bias in observational studies. In: *Observational studies.* Springer; 2002:71–104.

25. Keele L. An overview of rbounds: An R package for Rosenbaum bounds sensitivity analysis with matched data. *White Paper Columbus, OH.* 2010;**1**:15.

For peer review only

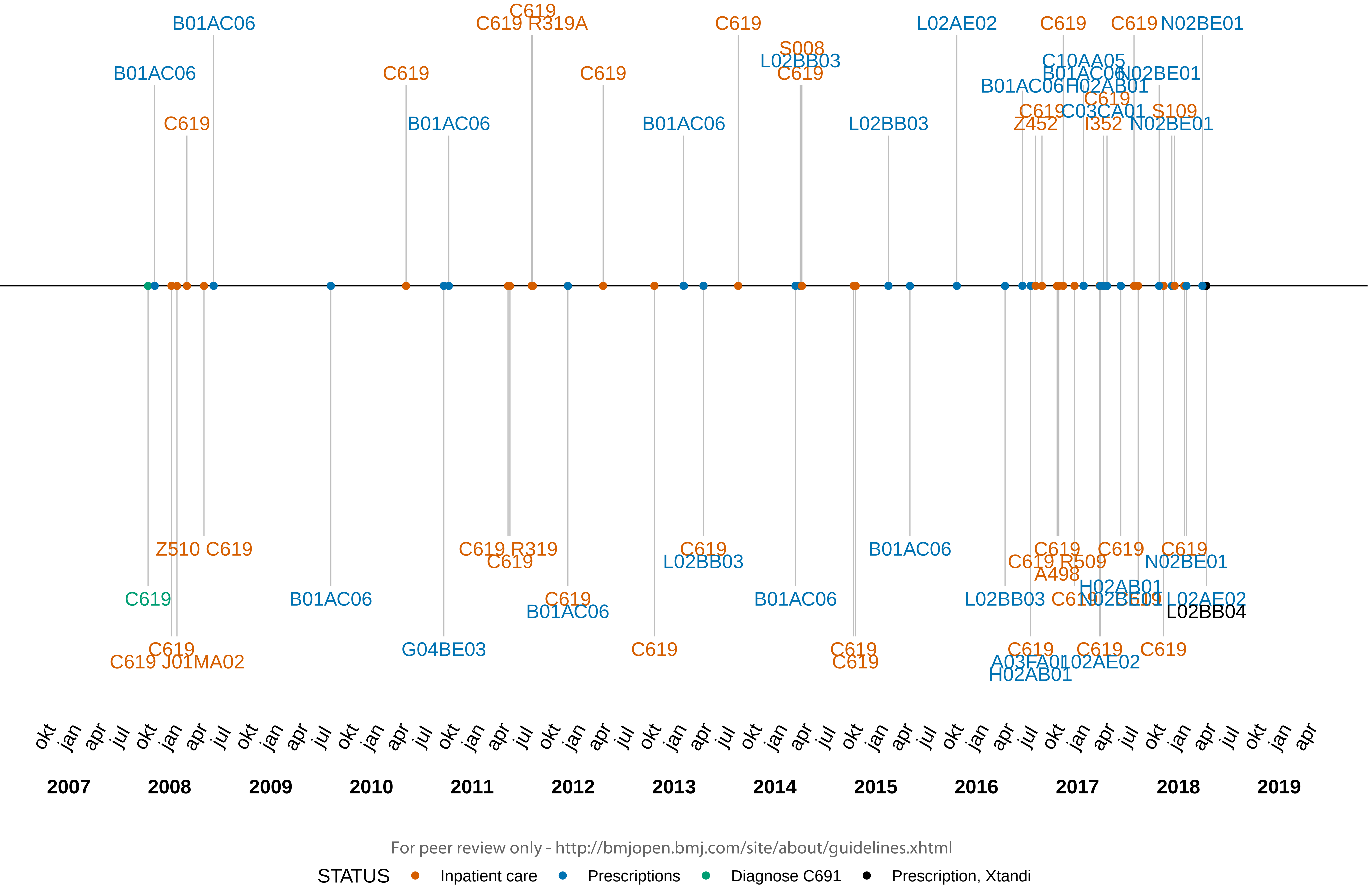


For peer review only - http://bmjopen.bmj.com/site/about/guidelines.xhtml

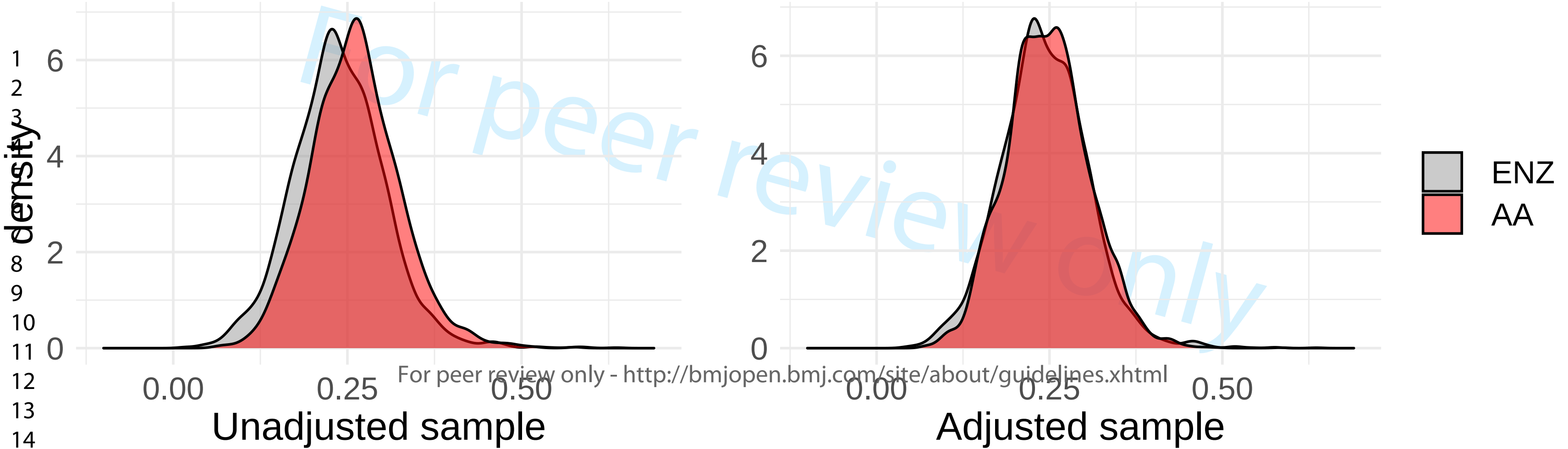


For peer review only

For peer review only - http://bmjopen.bmj.com/site/about/guidelines.xhtml

Figure S1: The fraction (the number of diagnoses divided by the number of men in the population) with a prostate cancer diagnose.

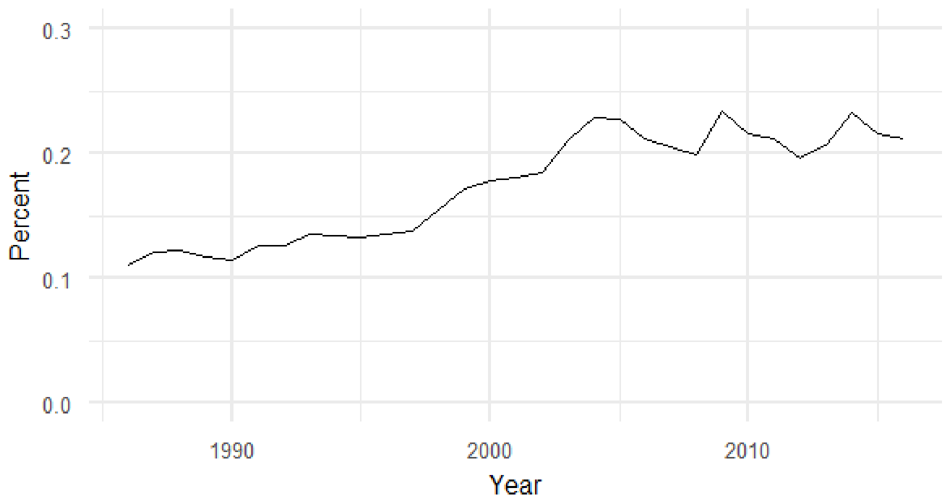

Table S1: Number of collected first time prescriptions per year after the subsidization, by year of prescription.

| Year | Enzalutamide | Abiraterone | Total |
|---|---|---|---|
| 2014 | 0 | 2 | 2 |
| 2015 | 864 | 407 | 1271 |
| 2016 | 1158 | 233 | 1391 |
| 2017 | 1005 | 321 | 1326 |
| 2018 | 454 | 157 | 611 |
| Total | 3481 | 1120 | 4601 |

For peer review only

Table S2: ICD codes used to specify related diagnoses

| Cardiovascular disease | Diabetes | Osteoporosis | Metastases | Malaise and fatigue |
|---|---|---|---|---|
| I21 | E10 | M859 | C77 | R53 |
| I22 | E11 | M810 | C78 | |
| I252 | E12 | M818 | C79 | |
| Z958 | E13 | M819 | | |
| Z959 | E14 | | | |
| I70 | | | | |
| I71 | | | | |
| I731 | | | | |
| I738 | | | | |
| I739 | | | | |
| I771 | | | | |
| I790 | | | | |
| I792 | | | | |
| K551 | | | | |
| K558 | | | | |
| K559 | | | | |
| I090 | | | | |
| I110 | | | | |
| I13 | | | | |
| I130 | | | | |
| I131 | | | | |
| I132 | | | | |
| I139 | | | | |
| I42 | | | | |

| | | | | |
|---|---|---|---|---|
| I420<br>I421<br>I422<br>I423<br>I424<br>I425<br>I426<br>I427<br>I428<br>I429<br>I43<br>I44<br>I45<br>I46<br>I47<br>I48<br>I49<br>I50<br>I51<br>R00 | | | | |

Table S3: All variables with description (* variable is included in factor analysis)

| Variable | Description |
|---|---|
| LOPNR | ID serial number |
| group | Treatment group (group=1 for abiraterone) |
| lan_namn | County council |
| h_tot_ov_bfd_i | Indicator of other CVD before diagnosis |
| h_tot_ov_bw_i | Indicator of other CVD between diagnosis and treatment |
| d_tot_bfd_i | Indicator of diabetes before diagnosis |
| d_tot_bw_i | Indicator of diabetes between diagnosis and treatment |
| o_tot_bfd_i | Indicator of osteoporosis before diagnosis |
| o_tot_bw_i | Indicator of osteoporosis between diagnosis and treatment |
| c_tot_bfd_i | Indicator of metastases before diagnosis |
| c_tot_bw_i | Indicator of metastases between diagnosis and treatment |
| f_tot_bfd_i | Indicator of fatigue before diagnosis |
| f_tot_bw_i | Indicator of fatigue between diagnosis and treatment |
| h_tot_ov_i | Indicator of other CVD before treatment |
| d_tot | Indicator of diabetes before treatment |

For peer review only

| | | |
|---|---|---|
| | o_tot | Indicator of osteoporosis before treatment |
| | c_tot | Indicator of metastases before treatment |
| | i48 | Indicator of atrial fibrillation and flutter before treatment |
| | i21 | Indicator of acute myocardial infarction before treatment |
| | i48_bfd_i | Indicator of atrial fibrillation and flutter before diagnosis |
| | i21_bfd_i | Indicator of acute myocardial infarction before diagnosis |
| | i48_bw_i | Indicator of atrial fibrillation and flutter before diagnosis |
| | i21_bw_i | Indicator of acute myocardial infarction before diagnosis |
| | f_tot | Indicator of fatigue before treatment |
| | h_tot | Indicator of any CVD before treatment |
| | Diff_time | Time between diagnosis and treatment |
| ‘ | bfd_1m | Number of visits, 1 month before diagnosis |
| ‘ | bfd_12m | Number of visits, 12 months before diagnosis |
| ‘ | bfd_60m | Number of visits, 5 years before diagnosis |
| ‘ | afd_1m | Number of visits, 0-1 month after diagnosis |
| ‘ | afd_2m | Number of visits, 1-2 months after diagnosis |
| ‘ | afd_3m | Number of visits, 2-3 months after diagnosis |
| ‘ | bft_1m | Number of visits, 0-1 month before treatment |
| ‘ | bft_2m | Number of visits, 1-2 months before treatment |
| ‘ | bft_3m | Number of visits, 2-3 months before treatment |
| ‘ | bft_4m | Number of visits, 3-4 months before treatment |
| ‘ | bft_5m | Number of visits, 4-5 months before treatment |
| ‘ | bft_12m_tot | Number of visits, 1 year before treatment |
| ‘ | bft_12m_tot_c619 | Number of C61.9 related visits, 1 year before treatment |
| ‘ | h_bfd_12m | Number of heart related visits, 1 year before treatment |
| ‘ | h_bft_3m | Number of heart related visits, 3 months before treatment |
| ‘ | msv_days_bd | Number of days in inpatient care before diagnosis |
| ‘ | msv_days_c619 | Number of days in inpatient care related to C61.9 |
| ‘ | msv_tot_prop_c619 | Fraction of days in inpatient care related to C61.9 |
| ‘ | msv_days | Days in inpatient care between diagnosis and treatment |
| ‘ | msv_tot_prop | Fraction of days in inpatient care between diagnosis and treatment |
| ‘ | nn | Visits per day between diagnosis and treatment |
| | h_tot_bfd | Visits related to CVD before diagnosis |
| | h_tot_bw | Visits related to CVD between diagnosis and treatment |
| ‘ | d_tot_bfd | Visits related to diabetes before diagnosis |
| ‘ | d_tot_bw | Visits related to diabetes between diagnosis and treatment |
| ‘ | o_tot_bfd | Visits related to osteoporosis before diagnosis |
| ‘ | o_tot_bw | Visits related to osteoporosis between diagnosis and treatment |
| ‘ | c_tot_bfd | Visits related to metastases before diagnosis |
| ‘ | c_tot_bw | Visits related to metastases between diagnosis and treatment |
| ‘ | f_tot_bfd | Visits related to fatigue before diagnosis |
| ‘ | f_tot_bw | Visits related to fatigue between diagnosis and treatment |
| ‘ | i48_bfd | Visits related to atrial fibrillation and flutter before diagnosis |
| ‘ | i21_bfd | Visits related to acute myocardial infarction before diagnosis |

For peer review only

| | | |
|---|---|---|
| ‘ | h_tot_ov_bfd | Visits related to other CVD before diagnosis |
| ‘ | i48_bw | Visits related to atrial fibrillation and flutter between diagnosis and treatment |
| ‘ | i21_bw | Visits related to acute myocardial infarction between diagnosis and treatment |
| ‘ | h_tot_ov_bw | Visits related to other CVD between diagnosis and treatment |
| ‘ | q1_t | Number of inpatient care visits between diagnosis and treatment, quartile 1 |
| ‘ | q2_t | Number of inpatient care visits between diagnosis and treatment, quartile 2 |
| ‘ | q3_t | Number of inpatient care visits between diagnosis and treatment, quartile 3 |
| ‘ | q4_t | Number of inpatient care visits between diagnosis and treatment, quartile 4 |
| ‘ | n_heart_kalc | Number of prescriptions of ATC code C08 |
| ‘ | n_heart_beta | Number of prescriptions of ATC code C07 |
| ‘ | n_diab | Number of prescriptions of ATC code A10 |
| | Mort_medel | Historical county specific mortality in prostate cancer |
| | Fodelseland_EU28 | Country of birth |
| | ALDER | Age at treatment |
| | Utbn=ForGymn | Educational level at treatment : less than secondary school |
| | Utbn=Gymn | Educational levelat treatment : secondary school |
| | Utbn=EfterGymn | Educational level at treatment : more than secondary school |
| | Civil | Marital status at treatment (Civil=1 if partner) |
| ‘ | LoneInk.x | Wage income at treatment |
| ’ | InkFNetto | Income from business at treatment |
| ‘ | ForvErs | Wage income at treatment |
| ’ | KapInk | Capital income at treatment |
| ’ | DispInk04 | Disposable income at treatment |
| ’ | DispInkFam04 | Family disposable income at treatment |
| ‘ | ArbErs | Wage income at treatment |
| ’ | LoneInk_1y | Wage income one year before treatment |
| ‘ | InkFNetto_1y | Income from business one year before treatment |
| ‘ | KapInk_1y | Capital income one year before treatment |
| ‘ | DispInk04_1y | Disposable income one years before treatment |
| ‘ | DispInkFam04_1y | Family disposable income one years before treatment |
| ‘ | ForvErs_1y | Wage income one year before treatment |
| ‘ | LoneInk_2y | Wage income two years before treatment |
| ‘ | InkFNetto_2y | Income from business two years before treatment |
| ‘ | ForvErs_2y | Wage income two years before treatment |
| ‘ | KapInk_2y | Capital income two years before treatment |
| ‘ | DispInk04_2y | Disposable income two years before treatment |
| ‘ | DispInkFam04_2y | Family disposable income two years before treatment |
| ‘ | SjukRe | Sickness compensation at treatment |

For peer review only

| | | |
|---|---|---|
| ’ | ArbLos | Unemployment benefits at treatment |
| ’ | ForTid | Early retirement benefit at treatment |
| ‘ | SocInk | Social security benefits at treatment |
| ‘ | SocBidrPersF04 | Social security benefits at treatment |
| ‘ | SocBidrFam | Social security benefits of the family at treatment |
| ‘ | SjukRe_1y | Sickness compensation one year before treatment |
| ‘ | ArbLos_1y | Unemployment benefits one year before treatment |
| ‘ | ForTid_1y | Early retirement benefit one year before treatment |
| ‘ | SocInk_1y | Social security benefits one year before treatment |
| ‘ | SocBidrPersF04_1y | Social security benefits one year before treatment |
| ‘ | SocBidrFam_1y | Social security benefits of the family one year before treatment |
| ‘ | SjukRe_2y | Sickness compensation two years before treatment |
| ‘ | ArbLos_2y | Unemployment benefits two years before treatment |
| ‘ | ForTid_2y | Early retirement benefit two years before treatment |
| ‘ | SocInk_2y | Social security benefits two years before treatment |
| ‘ | SocBidrPersF04_2y | Social security benefits two years before treatment |
| ‘ | SocBidrFam_2y | Social security benefits of the family two years before treatment |
| ‘ | SumAldP03 | Old-age pensions at treatment |
| ‘ | SumTjP | Occupational pensions one year before treatment |
| ‘ | PrivPens | Private pensions two years before treatment |
| ‘ | SumAldP03_1y | Old-age pensions at treatment |
| ‘ | SumTjP_1y | Occupational pensions one year before treatment |
| ‘ | PrivPens_1y | Private pensions two years before treatment |
| ‘ | SumAldP03_2y | Old-age pensions at treatment |
| ‘ | SumTjP_2y | Occupational pensions one year before treatment |
| ‘ | PrivPens_2y | Private pensions two years before treatment |
| | ALDER_D | Age at diagnosis |
| | Civil_D | Marital status at diagnosis (Civil=1 if partner) |
| ‘ | LoneInk_D | Wage income at diagnosis |
| ’ | InkFNetto_D | Income from business at diagnosis |
| ’ | KapInk_D | Capital income at diagnosis |
| ’ | DispInk_D | Disposable income at diagnosis |
| ’ | DispInkFam_D | Family disposable income at diagnosis |
| ’ | LoneInk_1y_D | Wage income one year before diagnosis |
| ‘ | InkFNetto_1y_D | Income from business one year before diagnosis |
| ‘ | KapInk_1y_D | Capital income one year before diagnosis |
| ‘ | DispInk_1y_D | Disposable income one year before diagnosis |
| ‘ | DispInkFam_1y_D | Family disposable income one year before diagnosis |
| ‘ | LoneInk_2y_D | Wage income two years before diagnosis |
| ‘ | InkFNetto_2y_D | Income from business two years before diagnosis |
| ‘ | KapInk_2y_D | Capital income two years before diagnosis |
| ‘ | DispInk_2y_D | Disposable two years before diagnosis |
| ‘ | DispInkFam_2y_D | Family disposable income two years before diagnosis |
| ‘ | SjukRe_D | Sickness compensation at diagnosis |

For peer review only

| | | |
|---|---|---|
| ’ | ArbLos_D | Unemployment benefits at diagnosis |
| ’ | ForTid_D | Early retirement benefit at diagnosis |
| ‘ | SocInk_D | Social security benefits at diagnosis |
| ‘ | SocBidrPersF_D | Social security benefits at diagnosis |
| ‘ | SocBidrFam_D | Social security benefits of the family at diagnosis |
| ‘ | SjukRe_1y_D | Sickness compensation one year before diagnosis |
| ‘ | ArbLos_1y_D | Unemployment benefits one year before diagnosis |
| ‘ | ForTid_1y_D | Early retirement benefit one year before diagnosis |
| ‘ | SocInk_1y_D | Social security benefits one year before diagnosis |
| ‘ | SocBidrPersF_1y_D | Social security benefits one year before diagnosis |
| ‘ | SocBidrFam_1y_D | Social security benefits of the family one year before diagnosis |
| ‘ | SjukRe_2y_D | Sickness compensation two years before diagnosis |
| ‘ | ArbLos_2y_D | Unemployment benefits two years before diagnosis |
| ‘ | ForTid_2y_D | Early retirement benefit two years before diagnosis |
| ‘ | SocInk_2y_D | Social security benefits two years before diagnosis |
| ‘ | SocBidrPersF_2y_D | Social security benefits two years before diagnosis |
| ‘ | SocBidrFam_2y_D | Social security benefits of the family two years before diagnosis |
| ‘ | AldPens_D | Old-age pensions at diagnosis |
| ‘ | SumTjP_D | Occupational pensions one year before diagnosis |
| ‘ | PrivPens_D | Private pensions two years before diagnosis |
| ‘ | AldPens_1y_D | Old-age pensions at diagnosis |
| ‘ | SumTjP_1y_D | Occupational pensions one year before diagnosis |
| ‘ | PrivPens_1y_D | Private pensions two years before diagnosis |
| ‘ | AldPens_2y_D | Old-age pensions at diagnosis |
| ‘ | SumTjP_2y_D | Occupational pensions one year before diagnosis |
| ‘ | PrivPens_2y_D | Private pensions two years before diagnosis |

Table S4: Estimated factor loadings

| | F1 | F2 | F3 | F4 | F5 | F6 | F7 | F8 | F9 |
|---|---|---|---|---|---|---|---|---|---|
| SocBidrPers_1y_D | 0.94 | | | | | | | | |
| SocBidrPers_D | 0.93 | | | | | | | | |
| SocBidrFam_D | 0.92 | | | | | | | | |
| SocBidrPers_2y_D | 0.91 | | | | | | | | |
| SocBidrFam_2y_D | 0.90 | | | | | | | | |
| SocBidrFam_1y_D | 0.90 | | | | | | | | |
| SocBidrPersF04_2y | 0.88 | | | | | | | | |
| SocBidrFam_2y | 0.87 | | | | | | | | |
| SocBidrPersF04_1y | 0.87 | | | | | | | | |
| SocBidrFam | 0.86 | | | | | | | | |
| SocBidrPersF04 | 0.85 | | | | | | | | |
| SocBidrFam_1y | 0.84 | | | | | | | | |
| LoneInk_1y | | 0.95 | 0.21 | | | | | | |

For peer review only

| | | | | | | | |
|---|---|---|---|---|---|---|---|
| LoneInk.x | 0.95 | | | | | | |
| ForvErs_1y | 0.94 | 0.22 | | | | | |
| ForvErs | 0.94 | | | | | | |
| LoneInk_2y | 0.92 | 0.25 | | | | | |
| ForvErs_2y | 0.91 | 0.27 | | | | | |
| LoneInk_1y_D | 0.60 | 0.58 | | | | | -0.37 |
| LoneInk_D | 0.59 | 0.56 | | | | | -0.37 |
| LoneInk_2y_D | 0.44 | 0.62 | | | | | -0.38 |
| DispInk04 | 0.31 | 0.37 | | | | | |
| DispInk04_1y | 0.25 | 0.34 | | | | | |
| DispInk_D | 0.25 | 0.52 | | | | | |
| DispInkFam04_1y | 0.21 | 0.31 | | | | | |
| SumAldP03 | -0.30 | | | -0.44 | | | 0.27 |
| SumAldP03_2y | -0.31 | | | -0.43 | | | 0.29 |
| SumAldP03_1y | -0.32 | | | -0.45 | | | 0.29 |
| SumTjP_1y | | 0.87 | | | | | 0.31 |
| SumTjP | | 0.85 | | | | | 0.29 |
| SumTjP_2y | | 0.84 | | | | | 0.33 |
| SumTjp_D | | 0.58 | | | | | 0.70 |
| SumTjp_1y_D | | 0.49 | | | | | 0.78 |
| SumTjp_2y_D | | 0.39 | | | | | 0.78 |
| AldPens_D | | 0.34 | | -0.20 | 0.38 | | 0.75 |
| DispInk_2y_D | | 0.32 | | | | | |
| DispInkFam_D | | 0.30 | | | | | |
| DispInk_1y_D | | 0.30 | | | | | |
| PrivPens | | 0.29 | | | 0.59 | | |
| DispInkFam_2y_D | | 0.26 | | | | | |
| DispInkFam04_2y | | 0.25 | | | | | |
| DispInkFam_1y_D | | 0.25 | | | | | |
| DispInk04_2y | | 0.25 | | | | | |
| PrivPens_2y | | 0.24 | | | 0.92 | | |
| PrivPens_1y | | 0.24 | | | 0.91 | | |
| AldPens_1y_D | | 0.24 | | | 0.38 | | 0.80 |
| DispInkFam04 | | 0.23 | | | | | |
| ForTid_1y_D | | | 0.90 | | | | |
| ForTid_D | | | 0.88 | | | | |
| SocInk_1y_D | | | 0.85 | | | | |
| ForTid_2y_D | | | 0.82 | | | | |
| SocInk_2y_D | | | 0.81 | | | | |
| SocInk_D | | | 0.71 | 0.28 | | | |
| ForTid_2y | | | 0.64 | | | | |
| ForTid_1y | | | 0.61 | | | | |
| ForTid | | | 0.57 | | | | |
| SocInk_2y | | | 0.52 | 0.40 | | | |
| SocInk_1y | | | 0.44 | 0.63 | | | |
| SocInk | | | 0.29 | 0.88 | | | |
| SjukRe_2y_D | | | 0.26 | | | | |
| AldPens_2y_D | | | -0.20 | | 0.38 | | 0.79 |
| ArbErs | | | | 0.96 | | | |
| SjukRe | | | | 0.96 | | | |

For peer review only

| | | | | |
|---|---|---|---|---|
| SjukRe_1y | 0.74 | | | |
| SjukRe_2y | 0.49 | | | |
| SjukRe_D | 0.45 | | | |
| nn | 0.21 | | 0.42 | |
| PrivPens_1y_D | | 0.94 | | |
| PrivPens_D | | 0.94 | | |
| PrivPens_2y_D | | 0.94 | | |
| bft_12m_tot | | | 0.92 | |
| bft_12m_tot_c619 | | | 0.61 | |
| bft_3m | | | 0.60 | |
| bft_4m | | | 0.59 | |
| bft_5m | | | 0.58 | |
| bft_2m | | | 0.57 | |
| q4_t | | | 0.53 | |
| bft_1m | | | 0.52 | |
| q3_t | | | 0.44 | |
| c_tot_bw | | | 0.35 | |
| msv_days_c619 | | | 0.34 | |
| q2_t | | | 0.34 | |
| msv_tot_prop | | | 0.33 | |
| bfd_12m | | | 0.33 | |
| bfd_60m | | | 0.33 | |
| msv_days | | | 0.33 | |
| msv_tot_prop_c619 | | | 0.30 | |
| h_bft_3m | | | 0.25 | |
| q1_t | | | 0.24 | |
| h_tot_bw | | | 0.22 | |
| InkFNetto_1y | | | | 0.97 |
| InkFNetto_2y | | | | 0.96 |
| InkFNetto | | | | 0.95 |
| InkFNetto_1y_D | | | | 0.73 |
| InkFNetto_2y_D | | | | 0.71 |
| InkFNetto_D | | | | 0.70 |

For peer review only

Table S5: Factor analysis results

| **Factor** | **Interpretation** | **Cumulative Variance** |
|---|---|---|
| Factor 1 | Welfare and social security benefits | 0.07 |
| Factor 2 | Wages and disposable income | 0.13 |
| Factor 3 | Occupational pensions | 0.18 |
| Factor 4 | Early retirement benefits and welfare | 0.22 |
| Factor 5 | Sickness and unemployment benefits | 0.27 |
| Factor 6 | Private pensions | 0.31 |
| Factor 7 | Health before treatment | 0.34 |
| Factor 8 | Income at diagnosis | 0.38 |
| Factor 9 | Income from business | 0.42 |

Table S6: LASSO, propensity score estimation

| Variable | Coeff. |
|---|---|
| d_tot1:ALDER_poly | -0.00005 (0.00003) |
| c_tot1:Diff_time_poly | 0.000 (0.000) |
| c_tot1:mort_medel_poly | 0.0001*** (0.00003) |
| i211:ALDER | 0.004 (0.021) |
| i211:Civil1 | -0.203 (0.318) |
| i211:ALDER_poly | -0.0001 (0.0003) |
| i481:Diff_time | -0.0001 (0.0001) |
| i481:ALDER_T_poly | 0.00005 (0.00004) |
| i481:Diff_time_poly | 0.000 (0.00000) |
| f_tot1:UTBNFORGYMN | -0.767** (0.312) |
| Civil1:mort_medel_poly | 0.00004 (0.00003) |
| d_tot_bfd_i1 | -0.191 (0.212) |
| c_tot_bfd_i1 | -0.227 (0.233) |
| DispInk04 | -0.00001 (0.00001) |
| ArbErs | -0.00000 (0.00000) |
| PrivPens | -0.0002** (0.0001) |
| SumAldP03_1y | 0.0001 (0.0001) |
| bfd_1m | -0.038 (0.045) |
| afd_3m | -0.062 (0.055) |
| c_tot_bw | 0.018*** (0.005) |
| i21_bfd | -0.111 (0.113) |
| msv_days_bd | 0.005* (0.003) |
| msv_tot_prop_c619 | -5.181* (2.665) |
| q1_t | -0.010** (0.005) |
| n_heart_kalc | 0.116* (0.065) |
| n_diab | -0.048 (0.057) |
| SjukRe_D | -0.0002 (0.0002) |
| SocInk_1y_D | 0.0002** (0.0001) |

For peer review only

BMJ Open

# A study protocol for a comparative effectiveness evaluation of Abiraterone Acetate against Enzalutamide: A longitudinal study based on Swedish administrative registers

| | |
|---|---|
| Journal: | *BMJ Open* |
| Manuscript ID | bmjopen-2021-052610.R1 |
| Article Type: | Protocol |
| Date Submitted by the Author: | 26-Sep-2021 |
| Complete List of Authors: | Johansson, Per; Uppsala University, Statistics; Tsinghua University, YMSC<br>Jonéus, Paulina; Uppsala University, Department of Statistics<br>Langenskiöld, Sophie; Uppsala University, Department of Medical Sciences |
| <b>Primary Subject Heading</b>: | Evidence based practice |
| Secondary Subject Heading: | Epidemiology, Oncology, Public health, Research methods, Urology |
| Keywords: | Protocols & guidelines < HEALTH SERVICES ADMINISTRATION & MANAGEMENT, EPIDEMIOLOGY, Epidemiology < ONCOLOGY, Urological tumours < ONCOLOGY, PUBLIC HEALTH, Prostate disease < UROLOGY |
| | |

SCHOLARONE™
Manuscripts

*I, the Submitting Author has the right to grant and does grant on behalf of all authors of the Work (as defined in the below author licence), an exclusive licence and/or a non-exclusive licence for contributions from authors who are: i) UK Crown employees; ii) where BMJ has agreed a CC-BY licence shall apply, and/or iii) in accordance with the terms applicable for US Federal Government officers or employees acting as part of their official duties; on a worldwide, perpetual, irrevocable, royalty-free basis to BMJ Publishing Group Ltd ("BMJ") its licensees and where the relevant Journal is co-owned by BMJ to the co-owners of the Journal, to publish the Work in this journal and any other BMJ products and to exploit all rights, as set out in our licence.*

*The Submitting Author accepts and understands that any supply made under these terms is made by BMJ to the Submitting Author unless you are acting as an employee on behalf of your employer or a postgraduate student of an affiliated institution which is paying any applicable article publishing charge ("APC") for Open Access articles. Where the Submitting Author wishes to make the Work available on an Open Access basis (and intends to pay the relevant APC), the terms of reuse of such Open Access shall be governed by a Creative Commons licence – details of these licences and which Creative Commons licence will apply to this Work are set out in our licence referred to above.*

*Other than as permitted in any relevant BMJ Author's Self Archiving Policies, I confirm this Work has not been accepted for publication elsewhere, is not being considered for publication elsewhere and does not duplicate material already published. I confirm all authors consent to publication of this Work and authorise the granting of this licence.*

For peer review only

# A study protocol for a comparative effectiveness evaluation of Abiraterone Acetate against Enzalutamide: A longitudinal study based on Swedish administrative registers

Per Johansson, Ph.D.[1,2]; Paulina Jonéus[1]; Sophie Langenskiöld, Ph.D[3]

1. Department of Statistics, Uppsala University
2. YMSC, Tsinghua University
3. Department of Medical Sciences, Uppsala University

Address for correspondence

Sophie Langenskiöld, PhD

Department of Medical Sciences, Uppsala University

Sophie.langenskiold@medsci.uu.se +46(0)709 393733

For peer review only

## Abstract

**Introduction:** This paper presents a study protocol for a comparative effectiveness evaluation of abiraterone acetate against enzalutamide in clinical practice, two cancer drugs given to patients suffering from advanced prostate cancer. **Method and analysis:** The protocol designs a comparative-effectiveness analysis of abiraterone acetate against enzalutamide. With the substantial number of covariates a two-step procedure is suggested in choosing relevant covariates in the matching design. In the first step, an exploratory factor analysis reduces the dimension of a large set of continuous covariates to nine factors. In the second step, we reduce the dimension of the covariates, interactions and second order terms for the continuous covariates using propensity score estimation. The final design makes use of a genetic matching algorithm. The study protocol provides a detailed statistical analysis plan of the analysis sample derived from the matching design. The analysis will make use of linear regression and robust inference adjusted for multi-significance testing. **Discussion:** As in a randomized experiment the focus is on the design of the assignment to treatment. This allows the publication of this pre-analysis plan before having access to outcome data. This means that the p-values will be correct if the maintained assumption of uncounfoundedness is valid. Given that p-hacking is substantial problem in empirical research, this is a substantial strength of the present study. However, while design yields, balance on the observed covariates one cannot discard the possibility that unobserved confounders are not balanced. For that reason, sensitivity tests for the maintained assumption of uncounfoundedness are presented. **Ethics and dissemination:** The study was approved by the Regional Ethical Review Board in Uppsala, Sweden (Dnr 2017/482). Results will be published in a peer-reviewed journal and distributed to relevant stakeholders in healthcare.


For peer review only

**Keywords:** protocol, comparative effectiveness research, real world evidence, metastatic castration-resistent prostate cancer, abiraterone acetate, enzalutamide, mortality, and morbidity.

## Strengths and limitations of this study:

- The study uses up-to-date mixed methods to design an observational study for a comparative effectiveness study of abiraterone acetate against enzalutamide of four outcomes.
- The study replicate a randomized study ex-post, by achieving comparable groups on patients' historical health and socioeconomic status based on rich linked population registries.
- As outcome data will be added after the publication of the protocol; the design and analysis are consequently not affected by post-treatment variables.
- Sensitivity analyses to evaluate potential remaining confounding are suggested in the protocol.

## Introduction

Prostate cancer (PC) is the most commonly diagnosed form of cancer in Sweden. In 2016, for example, 10,473 additional patients were diagnosed with prostate cancer creating a pool of 107,752 patients.[1,2] PC is also the second leading cause of cancer death, and almost all mortalities arise when the patients have progressed to the advanced stage, metastatic castrate resistant prostate cancer (mCRPC).

Various treatment alternatives are available for patients with mCRPC. In the last two decades, chemotherapy and novel hormone therapy (NHT) medications have revolutionized the treatment in mCRPC patients.[3-8] This paper presents a study protocol for the design of a comparative effectiveness evaluation of two of these NHTs, i.e. abiraterone acetate in

For peer review only

combination with prednisone (AA) and Enzalutamide (ENZ). The evaluation concerns their use in clinical practice from June 2015, which corresponds to the period when these drugs where reimbursed for mCRPC patients.

Data is collected from population registers administrated by the National Board of Health and Welfare (NBHW), and Statistics Sweden (SCB). The population is restricted to all men in the NBHW register with a prostate cancer diagnose before 2017 as only these patients were expected to progress to mCRPC during the period for which we planned to have outcome data. We restrict the population to patients prescribed AA or ENZ during the period June 1 2015 to June 15 2018, i.e. the period in which AA and ENZ were only prescribed for mCRPC.

The aim of the design is to estimate the comparative effectiveness of AA against ENZ using detailed data from the population registers and up to date statistical methods combined with an understanding of the prescription pattern of the patients obtained from a qualitative study with twelve urologists and oncologists at different hospitals and across different specialization all over Sweden. The main outcome of interest is overall mortality. Mortality data will be added after the publication of this study design. In addition, the comparative effectiveness on two secondary outcomes (pain and skeleton related events) will be studied. Treatment length and prostate specific mortality will be added as exploratory endpoints.

## Method and analysis

- Study population

This nationwide longitudinal study uses patient-level data from multiple population registries administrated by NBHW and SCB. Data from these registries are linked using a unique serial number created by SCB.

The population under study is defined using the cancer register. We first identify the number of men with a prostate cancer diagnosis before 2017 and the year of their diagnosis. The

For peer review only

annual number of men with diagnoses as a proportion of the men in the population has increased more than 100% between 1968 to 2001, from just above 0,1% in 1986 to more than 0,2% in 2016 (see Figure S1 in Appendix). We identify 243,535 unique patients with prostate cancer (ICD10 code C61.9 or earlier codes ICD7 177 and ICD9 185.9). If a patient for some reason has been diagnosed multiple times with this diagnosis, the first observed time period is used (1,661 patients appear multiple times, 1,623 of these diagnosed on two or more different dates and 181 patients are diagnosed at different hospitals).

The population is restricted to all men collecting a prescription of AA or ENZ during the period June 1 2015 to June 15 2018. The reasons for the time restriction are: (i) that almost no one was treated with these drugs before the reimbursement of AA and ENZ in June and July 2015, respectively, and (ii) that AA was additionally reimbursed in combination with ADT in patients with high-risk castration sensitive prostate cancer (mHSPC) in June 15 2018.

The restriction leaves us with a total of 4,601 patients in the study population. Some of the patients were prescribed AA or ENZ before the subsidization but collected the drug after June 2015, these are included in the sample. For this population the year of the diagnosis ranges between 1986 and 2016. Consequently, there is substantial variation in the time to be prescribed AA or ENZ from the date of diagnosis. This, so called, time to prescription is most likely an important covariate.

About 10% of the patients were prescribed both AA and ENZ over the years. We allocate these patients to the two samples AA and ENZ-takers based on their first prescription of one of the two drugs which means that the results should be interpreted as an intention to treat analysis. The number of AA and ENZ patients is increasing until 2016 (See Table S1 in the appendix).

For peer review only

The prescription of the two drugs varies over the 21 County councils, here after denoted counties, the responsible body for health care in Sweden. The proportions of AA and ENZ for the periods 2015 to 2018 across the counties are presented in Table 1. The fact that the prescription varies over counties is a notable finding as it suggests differences in prescription that may for instance be driven by practice variation. From this table we can furthermore see that in average across the counties, 24% of the patients were prescribed AA.

| | **Enzalutamide** | | | | | **Abiraterone** | | | | |
|---|---|---|---|---|---|---|---|---|---|---|
| County | 2015 | 2016 | 2017 | 2018 | TOT | 2015 | 2016 | 2017 | 2018 | TOT |
| Blekinge | 0.84 | 0.91 | 0.84 | 0.80 | 0.85 | 0.16 | 0.09 | 0.16 | 0.20 | 0.15 |
| Dalarna | 0.86 | 0.65 | 0.55 | 0.62 | 0.69 | 0.14 | 0.35 | 0.45 | 0.38 | 0.31 |
| Gavleborg | 0.60 | 0.92 | 0.93 | 0.96 | 0.85 | 0.40 | 0.08 | 0.07 | 0.04 | 0.15 |
| Gotland | 0.75 | 0.92 | 1.00 | 1.00 | 0.89 | 0.25 | 0.08 | | | 0.11 |
| Halland | 0.75 | 0.89 | 0.88 | 0.76 | 0.82 | 0.25 | 0.11 | 0.12 | 0.24 | 0.18 |
| Jamtland | 0.47 | 0.81 | 0.77 | 0.67 | 0.70 | 0.53 | 0.19 | 0.23 | 0.33 | 0.30 |
| Jonkopings lan | 0.62 | 0.91 | 0.89 | 0.76 | 0.80 | 0.38 | 0.09 | 0.11 | 0.24 | 0.20 |
| Kalmar | 0.74 | 0.89 | 0.83 | 0.93 | 0.84 | 0.26 | 0.11 | 0.17 | 0.07 | 0.16 |
| Kronoberg | 0.48 | 0.43 | 0.21 | 0.20 | 0.37 | 0.52 | 0.57 | 0.79 | 0.80 | 0.63 |
| Norrbotten | 0.83 | 0.88 | 0.64 | 0.65 | 0.74 | 0.17 | 0.12 | 0.36 | 0.35 | 0.26 |
| Orebro | 0.77 | 0.95 | 0.68 | 0.38 | 0.74 | 0.23 | 0.05 | 0.32 | 0.62 | 0.26 |
| Ostergotlands lan | 0.12 | 0.90 | 0.88 | 0.90 | 0.83 | 0.88 | 0.10 | 0.12 | 0.10 | 0.17 |
| Skane | 0.87 | 0.96 | 0.96 | 0.85 | 0.92 | 0.13 | 0.04 | 0.04 | 0.15 | 0.08 |
| Sodermanland | 0.77 | 0.98 | 1.00 | 0.92 | 0.91 | 0.23 | 0.02 | | 0.08 | 0.09 |
| Stockholm | 0.71 | 0.82 | 0.80 | 0.79 | 0.78 | 0.29 | 0.18 | 0.20 | 0.21 | 0.22 |
| Uppsala | 0.62 | 0.56 | 0.25 | 0.36 | 0.46 | 0.38 | 0.44 | 0.75 | 0.64 | 0.54 |
| Varmland | 0.87 | 0.96 | 0.67 | 0.75 | 0.83 | 0.13 | 0.04 | 0.33 | 0.25 | 0.17 |
| Vasterbotten | 0.63 | 0.70 | 0.62 | 0.76 | 0.67 | 0.37 | 0.30 | 0.38 | 0.24 | 0.33 |
| Vasternorrland | 0.47 | 0.71 | 0.73 | 0.86 | 0.64 | 0.53 | 0.29 | 0.27 | 0.14 | 0.36 |
| Vastmanland | 0.73 | 0.79 | 0.80 | 0.55 | 0.74 | 0.27 | 0.21 | 0.20 | 0.45 | 0.26 |
| Vastra gotalands lan | 0.51 | 0.78 | 0.69 | 0.65 | 0.66 | 0.49 | 0.22 | 0.31 | 0.35 | 0.34 |

For peer review only

| | | | | | | | | | | |
|---|---|---|---|---|---|---|---|---|---|---|
| Total | 0.68 | 0.83 | 0.76 | 0.74 | 0.76 | 0.32 | 0.17 | 0.24 | 0.26 | 0.24 |

Table 1: Proportion prescribed enzalutamide and abiraterone acetate respectively, per county and in total.

- Linked registers

From the NBHW, we link data from the inpatient care register and the pharmaceutical register. All in- and outpatient care visits in Sweden and all prescribed drugs are registered in these registers. The inpatient care register contains among others information on all diagnoses (using the ICD classifications), the date of admission and discharge. The pharmaceutical register contains the date of prescribing and dispensing of drugs, and also, the ATC class of the drug.

From SCB, we link data from a census conducted every fifth year over the period 1960-2015; labor statistics based on administrative sources (RAMS) or LISA for the period 1985-2015. RAMS and LISA are large data bases, created by linking a large set of administrative registers using the Swedish person id in the linkage. The linked data contains disposable income, labor income, social insurance payments, capital income, labor market status, year of birth, education, marital status, etc. for each individual over the period 1960-2015.

- Confounding

Groups on the different interventions can only be compared in case they are identical on all those variables which simultaneously influence their prescriptions and outcomes. Therefore, we first conducted a qualitative study (Langenskiöld 2021, submitted for publication) with its primary focus of understanding who prescribed which of the drugs, when, and for how long.

The response from the interviews where heterogeneous. There is however quite good agreement on the following three statements that are relevant for this paper. Firstly, ENZ is preferred for patients with cardiovascular diseases, diabetes, or osteoporosis whereas AA is

For peer review only

preferred for patients with poor general health condition and fatigue. Secondly, doctors want their patients to benefit from as many treatment options as possible during their lifetime. If it turns out that patients' general health is good and the disease is progressing fast, chemotherapy is the preferred treatment. Instead, if it turns out that patients' general health is poor and the disease is progressing slowly, ENZ or AA is the preferred treatment. Thirdly, doctors monitor the treatment and if it is found that treatments were ineffective or intolerable, the treatment were stopped.

- Covariates

From the inpatient care registers and the pharmaceutical register, we have extensive historical information on patients' health and health care consumption. This enables us to create an almost infinite number of covariates. We use health data for the period up to five years before the diagnosis, and leave out health data from the date of treatment and onward. That is, all covariates are measured before the first prescription of AA or ENZ and can consequently not be affected by the two treatments.

To exemplify the available data, the medical history from the inpatient care and prescription registry of a randomly chosen patient is presented in Figure 1. This patient was diagnosed with a prostate cancer in October 2007 (i.e. a C61.9 ICD-10 code) and was first treated with ENZ in April 2018 (i.e. a L02BB04 ATC code). This patient had no inpatient care visits the years before the cancer diagnosis, and the visits after the diagnosis is mainly related to the prostate cancer diagnose. The patient was hospitalized for more than one day in a row at two occasions.

[Figure 1 about here]

The medical history data is aggregated separately over a five years period before the diagnosis and for the period in-between diagnosis and prescription. We derive, among others,

For peer review only

the number and length of health care visits over the two periods. This is also done separately for patients experiencing metastases, cardiovascular disease, diabetes, and fatigue or osteoporosis diagnosis. We also derive the number of specific drugs collected between the cancer diagnose and treatment separately for prescriptions related to these diagnoses.

To gain information on sickness progression, we separately calculate the number of visits the last months before treatment and calculate the average number of visits per quarter between diagnosis and treatment.

Using this strategy, we create 45 continuous covariates where 13 measure the health status before the prostate cancer diagnose. We also derive 22 indicator variables, of which 7 measure health status before diagnosis, of whether a patient have had a diagnosis related to metastases, cardiovascular disease, diabetes, fatigue or osteoporosis. See Table S2 in Appendix for the included ICD codes.

From the SCB data we create 94 variables that supposedly will be able to describe the socioeconomic status of the patient three years before both the diagnosis and the treatment, with data from 1991 until 2015. For patients with diagnoses before or after these years, data on socioeconomic status is taken from the year closest to the year of diagnosis. This includes information on age, marital status and educational level as well as pensions, income, sick leave and other security benefits for the patient and the household. In the case of missing values, the mean over the three preceding years is used. Educational level is the highest completed education and is classified as less than, equal or more than secondary school.

In addition to the 161 covariates we also add historical county specific mortality in prostate cancer. That is, the mean number of men per 100,000 inhabitants who died from prostate cancer in 1997-2017. All 162 covariates with descriptions are presented in Table S3 in Appendix. Table 2 provides summary statistics of the AA and ENZ patients for the subset of

For peer review only

covariates deemed to be the most important. From this table we can see that the two groups in general are very similar, there are for instance no significant differences in average age, educational level or marital status.

| **Description** | **ENZ** | **AA** | **Diff** |
|---|---|---|---|
| Age at treatment | 75.27 (7.85) | 75.29 (7.70) | -0.02 |
| Years to treatment from diagnosis | 6.95 (5.00) | 7.29 (5.31) | 0.34* |
| Less than secondary school education | 0.36 (0.48) | 0.35 (0.48) | 0.01 |
| Secondary school education | 0.39 (0.49) | 0.39 (0.49) | -0.00 |
| More than secondary school education | 0.25 (0.43) | 0.26 (0.44) | -0.01 |
| Living with a partner | 0.66 (0.47) | 0.67 (0.47) | -0.01 |
| Cardiovascular disease before treatment | 0.35 (0.48) | 0.34 (0.47) | 0.01 |
| Atrial fibrillation and flutter before treatment (I48) | 0.16 (0.36) | 0.18 (0.38) | -0.02 |
| Acute myocardial infarction before treatment (I21) | 0.10 (0.30) | 0.07 (0.25) | 0.03*** |
| Other cardiovascular diseases before treatment | 0.27 (0.45) | 0.26 (0.44) | 0.01 |
| Diabetes before treatment | 0.16 (0.37) | 0.12 (0.32) | 0.04*** |
| Osteoporosis before treatment | 0.01 (0.11) | 0.01 (0.10) | 0.00 |
| Secondary malignant neoplasms (metastases) | 0.70 (0.46) | 0.75 (0.43) | 0.05*** |
| Malaise and fatigue | 0.05 (0.22) | 0.04 (0.20) | 0.01 |

*Standard deviations within parentheses. * $p < 0.1$; ** $p < 0.05$; *** $p < 0.01$.*

Table 2: Summary statistics of the AA and ENZ patients for the subset of covariates deemed to be the most important.

For peer review only

## Statistical analysis plan

### - Design

A genetic matching approach is used in the design.[9] This is a generalization of Mahalanobis distance matching where an evolutionary search algorithm is used to maximize the balance of a set of observed covariates together with an estimated propensity score.[10]

Due to computational limitations, we cannot match on all covariates. Thus, we need to restrict the set to the ones deemed the most important. According to the twelve urologists and oncologists interviewed for this project, cardiovascular diseases, fatigue, diabetes, osteoporosis and age at treatment influenced their choice of prescriptions and were thus included. As prevalence of visceral metastases indicated the severity of the disease, and the sequencing of prostate-cancer drugs reflected the optimization of care according to the same specialists, we also considered presence of visceral metastases and time to prescription. Also socioeconomic variables such as educational level, marital status, and quality of care differences across counties (measured by county specific mortality) were included. We also include nine factors that summarize the information from the 130 remaining covariates and an estimated propensity score (see Table S4 in Appendix for the factor loading of the set of variables included in the factor analysis).

The factors are derived from an exploratory factor analysis (using the varimax rotation and factor scores derived using the regression method.[11,12] The nine factors account for 42 percent of the variance in the 130 variables.

The propensity score is estimated using a logit model, and LASSO regression.[13] All 162 covariates are included, in addition, interactions and second order polynomials of the continuous covariates are included. The LASSO regression has a penalty, or cost, of including too many covariates.  This helps us avoid the risk of over-parametrizing the propensity-score which may lead to bias.[14]

For peer review only

A one-to-one matching with replacement is used in the genetic matching approach, but we exclude observations with a distance above three standard deviations for any of the included covariates. This leads to 85 dropped observations and a total of 4,516 matched observations.

[Figure 2 about here]

The propensity score balance is presented in Figure 2. It is clear that the estimated probability to be treated is similar already in the unmatched groups, but the balance is improved in the adjusted sample. The covariate balance in the matched sample is examined through the standardized difference in percent of the average standard deviation (SB). Further, we examine the variance ratio (VR) between the two groups for each of the included covariates. In a well conducted randomized experiment, we expect the SB to be no larger than 25 percent and the VR to be close to one. This is used as a rule of thumb in the quasi experimental literature.[15] The results are presented in Table 3.

| | SB before | SB after | VR before | VR after |
|---|---|---|---|---|
| Age (Years) | 0.28 | 1.10 | 0.96 | 0.90 |
| Diabetes (Y/N) | 12.48 | 0.70 | 0.77 | 0.99 |
| Other CVD (Y/N) | 3.65 | 1.77 | 0.96 | 0.98 |
| Acute myocardial infarction, I21 (Y/N) | 10.99 | 0.00 | 0.72 | 1.00 |
| Atrial fibrillation and flutter, I48 (Y/N) | 5.43 | 0.91 | 1.10 | 0.98 |
| Osteoporosis (Y/N) | 1.79 | 0.00 | 0.85 | 1.00 |
| Fatigue (Y/N) | 4.56 | 0.00 | 0.82 | 1.00 |
| Less than secondary school education | 3.15 | 5.08 | 0.98 | 1.03 |
| Secondary school education | 0.45 | 2.32 | 1.00 | 0.99 |
| Time to treatment (Years) | 6.58 | 0.55 | 1.13 | 0.94 |
| Secondary malignant neoplasms (Y/N) | 11.73 | 1.48 | 0.89 | 0.99 |
| Time to treatment squared (Years) | 7.75 | 1.26 | 1.23 | 0.88 |
| Age squared (Years) | 0.07 | 0.58 | 0.97 | 0.91 |
| Age and Diabetes | 12.41 | 0.45 | 0.77 | 1.00 |
| Age and CVD | 3.44 | 1.43 | 0.97 | 0.99 |
| Age and Secondary malignant neoplasms | 12.06 | 1.59 | 0.90 | 0.98 |
| Diabetes and CVD | 6.77 | 0.00 | 0.77 | 1.00 |

For peer review only

| | | | | |
|---|---|---|---|---|
| County specific mortality | 6.05 | 0.23 | 0.99 | 0.90 |
| Partner (Y/N) | 4.01 | 6.33 | 0.94 | 0.90 |
| Factor 1 | 1.03 | 1.48 | 1.08 | 0.72 |
| Factor 2 | 1.29 | 0.54 | 0.36 | 0.94 |
| Factor 3 | 0.66 | 5.03 | 0.68 | 0.81 |
| Factor 4 | 5.07 | 2.39 | 1.42 | 0.94 |
| Factor 5 | 4.37 | 1.48 | 0.71 | 0.95 |
| Factor 6 | 5.54 | 3.35 | 0.13 | 0.76 |
| Factor 7 | 1.30 | 1.90 | 0.53 | 0.78 |
| Factor 8 | 0.15 | 0.43 | 0.90 | 0.90 |
| Factor 9 | 0.21 | 0.35 | 0.48 | 0.97 |
| Propensity score | 37.03 | 9.10 | 0.76 | 0.83 |

Table 3: Standardized difference in percent and variance ratio before and after matching

- Pre-analysis plan

We have one primary outcome, two secondary outcomes and one exploratory outcome. The primary outcome is all-cause mortality (DEAD) and the two secondary outcomes capturing morbidity are PAIN, and SRE. PAIN is an indicator for severe pain, and SRE is an indicator for a skeleton related events.

The reason for including PAIN and SRE as outcomes is that these two morbidities have been seen as common complications of bone metastases.[16,17] The reason for studying COMPLY as an exploratory outcome is that it captures the treatments' ability to remain effective and/or safe. According to our qualitative work the main reasons for treatment interruptions is a lack of safety and being effective.

For the inference we will use Bonferroni correction with a five percent overall level. With one primary and two secondary outcomes this means that the significance level on the single outcomes will be 1.67% (= 100*0.05/3).

DEAD as a primary endpoint is an indicator variable defined as one for patients who are dead of any causes and zero for other patients at the end of each 30 days period after being

For peer review only

prescribed AA- or ENZ. Mortality data will be available until the end of June, 2020. Consequently, the first patient administered the treatments can theoretically have up to 70 mortality registrations, but the actual number of registrations will most likely be fewer as they are suffer a deadly disease.

Patients are assumed to suffer severe pain if they receive prescriptions for neuropathic pain, i.e., opiates in combination with tramadol and paracetamol (ATC-codes N02AA, N02AX02, and N02BE01). The PAIN indicator is one for periods in which the patient has received such a subscription and zero for the other periods. Prescription data will be available until 31st of December, 2020. Therefore, up to 76 periods are available for prescription data.

Patients are assumed to suffer skeleton related event if they experience a hospitalization because of pathologic fracture (ATC codes M485, M495, M844, and M907) or spinal cord compression (G550, G834, G952, G958, G959, and G992)[18]. The SRE indicator is one for periods with such hospitalizations and zero for the other periods. As inpatient care data is available until 31st of December, 2019, at most 64 periods are available.

For each of the periods, we will estimate the above three effects. We will display the Bonferroni adjusted confidence intervals (i.e. using the 1.67% level for an overall 5% level test) at each time period.

For all outcomes, regression analysis will be used in the estimation of the comparative effectiveness analyses of AA against ENZ. We adjust for all variables displayed in Table 2 except for the propensity score. In addition, we allow for heterogeneous effects by also adjusting for the covariates interacted with an indicator for AA. For the inference we will use the Eicker-Huber-White (EHW) covariance estimator robust covariance matrix. [19-21]

The problem with analysis of the two morbidity outcomes is that they are only observed in data if the patient is alive. In the analysis above we will, at each evaluation period, remove

For peer review only

the dead patients from the analysis. This means that the number of valid observations will be reduced over the evaluation window. If there are no differences in mortality rates between the two drugs this analysis strategy provides unbiased estimates of the comparative effectiveness on morbidity. However, if there are differences in mortality rates, these analyses are biased as it is more likely that we observe a morbidity for the drug with lower mortality rates. If this is the case, we will need to estimate bounds of potential effects as a sensitivity analysis. We let all patients who die either have the morbidity or not (i.e. PAIN =1 and SRE=1 and PAIN =0 and SRE=0). If the mortality is observed to be higher for AA than for ENZ the first case provides the upper bound estimate of the comparative effectiveness of AA against ENZ while the second one provides the lower bound on these two morbidity outcomes and vice versa if the mortality rate is lower for AA.

In addition, we will analyze two explanatory endpoints. The first is prostate-specific mortality and the second is the Duration of Reversing Treatments (DRT). Prostate-specific mortality is measure in 30 days' intervals in the same way as in the main analysis. DRT is measured as the duration until the patients is observed to have stopped their treatment. DRT is calculated based on prescription data and their average estimated compliance rate.

The problem with analyzing prostate-specific mortality is of how to deal with people dying from other causes of death. To this end we repeat the analysis with the two morbidity outcomes, that is, we remove patients who die from other causes and also estimate the upper and lower bound where those who die from other causes are either treated as dying from prostate cancer or not dying. All estimates are performed using the regression models explained above. The analysis of DRT will be conducted using Cox regression. Covariates displayed in Table 2 will be added in addition to the indicator for AA. We treat mortality as a competing risk conditional on the observed covariates.

For peer review only

- Subgroup analyses

Sub-analyses on these four outcomes will be conducted on patients who are believed (i) to be potential high-/low responders and; (ii) to suffer a more aggressive disease (iii) to have had a long or short time until subscription.

Using the results from our qualitative work we define high responder patients in (i) as those who are treated twelve months or more to previous hormone treatment i.e., LHRH agonists or –antagonists, while the low responder is treated shorter. The hormone treatments are Leuprolide (ATC L02AE02), Goserelin (ATC L02AE03), Triptorelin (ATC L02AE04), och Histrelin (ATC L02AE05) and Degarelix (ATC L02BX02), respectively. This corresponds to 63 percent of the sample being categorized as high respondents.

We regard patients with visceral metastases in their hospital records (ICD-10 code C78) before they start their AA or ENZ treatment in (ii) as having have more aggressive disease.[22,23] About seven percent of the patients are defined as suffering from a more aggressive disease.

All patients with a time between diagnosis of prostate cancer and prescription for AA or ENZ treatment shorter than the median waiting time for both drugs are defined as patients with short time until subscription (iii) while the complement is patients with long time.

- Sensitivity analyses

While the matching design yields balanced observed covariates, this is an observational study with the usual limitations in this context. In particular, one cannot discard the possibility that unobserved confounders are not balanced. However, Rosenbaum's sensitivity analysis for hidden bias will be used.[24] McNemar's test will be used when calculating ranges of p-values of the Intent to treat effect for different values of the sensitivity parameter.[25]

For peer review only

In addition, we will add data from the national prostate cancer register (NPCR). These data contain more detailed information on patient's heath with regard to the prostate cancer but do not have full coverage. The data is ordered and we will have access to it at the same time as we have the mortality data. These data allow us to basically test the validity of the design by estimating the effects on potential pre-treatment confounders. If there is a statically significant effect on these covariates this suggests that available data from population register are not sufficient to control for confounding bias.

For the test we use three covariates, that during discussions with specialists, are judged to be important confounders: PSA levels (D_SPSA), Gleason score (D_GleasSa), and metastases (D_Mstad) at the time of prostate cancer diagnoses. With three pre-measured covariates we, as in the main analysis, we adjust the significance level for the individual tests using Bonferroni correction based on a five percent overall level. This means that the level for testing on each single outcome is 1.67 percent. For all three covariates, regression analysis will be used in the estimation using the same regression designs as for the main analysis. NPCR also allows us to do more exploratory subgroup analyses. As one of the drug company we consulted believed that the initial treatment may influence the effectiveness of their drug, we will separately evaluate patients who receive conservative therapy (wait and see), radical prostatectomy, and radiation therapy as their primary treatment.

Generalizability of the results to other populations may not necessarily be granted, for instance, if future treated populations differ greatly in characteristics, which modify the effect of the treatment.

## Discussion

This protocol has specified a design and a detailed pre-analysis plan for a comparative effectiveness analysis using administrative registers. As in an RCT, the focus is on the design of the assignment mechanism before observing the outcome. The matching design creates an

For peer review only

analysis sample of the two treatment groups that are comparable on observed covariates as in an RCT. The approach lend objectivity to our results. Contrary to RCT, we can however not assure the two treatment groups are comparable on unobservable covariates. For that reason, sensitivity tests for the maintained assumption of unconfoundedness are presented in the protocol.

## Public and private involvement

Clinicians (Oncologists and Urologist) were involved in previous works for this protocol development. The clinicians were interviewed to understand factors that are important while prescribing AA and ENZ.

## Ethics and dissemination

Ethical approved by the Ethical committee in Uppsala (ref. Dnr2017/482). Results will be published in a peer-reviewed journal and distributed to healthcare providers, healthcare policymakers and potentially patients by way of printed and electronic materials as well as oral presentation at regional meetings.

## Author contribution

PJ and SL conceived the main conceptual ideas to replicate a randomized study ex-post using Swedish population registries. SL lead and planned the project, and carried out the preparatory qualitative work. PJ together with PaJ worked out the technical details in discussion with SL, and performed the analytical computations. All authors discussed the results and contributed to the final manuscript although PJ wrote the main part.

## Funding statement

This work was supported by the Dental and Benefit Agency [diary number 02823/2017]

## Competing interests statement

The authors declare that they have no competing interests.

For peer review only

## Word Count

4,046 excluding references.

## Acknowledgement

We would like to acknowledge the valuable contribution of the urologists and oncologists who participated in our qualitative work (R. Blom, G. Ahlgren, Å. Jellvert, M. Anden, K. Holmsten, S-O. Andersson, L. Nygren, L. Åström, M. Hellström, C. Thellenberg, J. Yachnin, J. Sandzen). They helped us understand what factors govern the prescription of AA, ENZ, i.e., factors which needs to balance in any groups compared in a comparative effectiveness evaluation. Also, we would like to thank the groups of experts with whom we intervened in order to improve our protocol, i.e., either the balance across the groups or the endpoints to focus on (A. Bill-Azelsson, D. Robinson, R. Henriksson, and representatives from Astellas Pharma and Janssen Inc, i.e., Kerstin Magnusson, Johanna Svensson and Ingrid Tredal and Julia Rockberg, respectively. Finally, we thank Sven-Åke Lööf for sharing his great understanding of the field.

## References

1. Fitzmaurice C, Abate D, Abbasi N, et al. Global, regional, and national cancer incidence, mortality, years of life lost, years lived with disability, and disability-adjusted life-years for 29 cancer groups, 1990 to 2017: a systematic analysis for the global burden of disease study. *JAMA oncology.* 2019;5(12):1749-1768.
2. Socialstyrelsen. Cancer i siffror 2018 populärvetenskapliga fakta om cancer, Cancerfonden och Socialstyrelsen i samarbete. 2018; https://www.socialstyrelsen.se/globalassets/sharepoint-dokument/artikelkatalog/statistik/2018-6-10.pdf Accessed October 15, 2019.
3. Basch E, Autio K, Ryan CJ, et al. Abiraterone acetate plus prednisone versus prednisone alone in chemotherapy-naive men with metastatic castration-resistant prostate cancer: patient-reported outcome results of a randomised phase 3 trial. *The lancet oncology.* 2013;14(12):1193-1199.
4. Beer TM, Armstrong AJ, Rathkopf DE, et al. Enzalutamide in metastatic prostate cancer before chemotherapy. *New England Journal of Medicine.* 2014;371(5):424-433.
5. Loriot Y, Miller K, Sternberg CN, et al. Effect of enzalutamide on health-related quality of life, pain, and skeletal-related events in asymptomatic and minimally symptomatic, chemotherapy-naive patients with metastatic castration-resistant

For peer review only

prostate cancer (PREVAIL): results from a randomised, phase 3 trial. *The Lancet Oncology.* 2015;16(5):509-521.

6. Ryan CJ, Smith MR, De Bono JS, et al. Abiraterone in metastatic prostate cancer without previous chemotherapy. *New England Journal of Medicine.* 2013;368(2):138-148.
7. Tannock IF, De Wit R, Berry WR, et al. Docetaxel plus prednisone or mitoxantrone plus prednisone for advanced prostate cancer. *New England Journal of Medicine.* 2004;351(15):1502-1512.
8. Ye D, Huang Y, Zhou F, et al. A phase 3, double-blind, randomized placebo-controlled efficacy and safety study of abiraterone acetate in chemotherapy-naïve patients with mCRPC in China, Malaysia, Thailand and Russia. *Asian journal of urology.* 2017;4(2):75-85.
9. Diamond A, Sekhon JS. Genetic matching for estimating causal effects: A general multivariate matching method for achieving balance in observational studies. *Review of Economics and Statistics.* 2013;95(3):932-945.
10. Sekhon JS, Mebane Jr WR. Genetic optimization using derivatives. *Political Analysis.* 1998:187-210.
11. Costello AB, Osborne J. Best practices in exploratory factor analysis: Four recommendations for getting the most from your analysis. *Practical assessment, research, and evaluation.* 2005;10(1):7.
12. DiStefano C, Zhu M, Mindrila D. Understanding and using factor scores: Considerations for the applied researcher. *Practical Assessment, Research, and Evaluation.* 2009;14(1):20.
13. Tibshirani R. Regression shrinkage and selection via the lasso. *Journal of the Royal Statistical Society: Series B (Methodological).* 1996;58(1):267-288.
14. De Luna X, Waernbaum I, Richardson TS. Covariate selection for the nonparametric estimation of an average treatment effect. *Biometrika.* 2011;98(4):861-875.
15. Imbens GW, Rubin DB. *Causal inference in statistics, social, and biomedical sciences.* Cambridge University Press; 2015.
16. Drudge-Coates L, Oh WK, Tombal B, et al. Recognizing symptom burden in advanced prostate cancer: a global patient and caregiver survey. *Clinical genitourinary cancer.* 2018;16(2):e411-e419.
17. So A, Chin J, Fleshner N, Saad F. Management of skeletal-related events in patients with advanced prostate cancer and bone metastases: Incorporating new agents into clinical practice. *Canadian Urological Association Journal.* 2012;6(6):465.
18. Parry MG, Cowling TE, Sujenthiran A, et al. Identifying skeletal-related events for prostate cancer patients in routinely collected hospital data. *Cancer epidemiology.* 2019;63:101628.
19. Eicker F. Limit theorems for regressions with unequal and dependent errors. Paper presented at: Proceedings of the fifth Berkeley symposium on mathematical statistics and probability1967.
20. Huber PJ. The behavior of maximum likelihood estimates under nonstandard conditions. Paper presented at: Proceedings of the fifth Berkeley symposium on mathematical statistics and probability1967.

For peer review only

21. White H. A heteroskedasticity-consistent covariance matrix estimator and a direct test for heteroskedasticity. *Econometrica: journal of the Econometric Society.* 1980:817-838.

22. Mazzone E, Preisser F, Nazzani S, et al. Location of metastases in contemporary prostate cancer patients affects cancer-specific mortality. *Clinical genitourinary cancer.* 2018;16(5):376-384. e371.

23. Whitney CA, Howard LE, Posadas EM, et al. In Men with Castration-Resistant Prostate Cancer, Visceral Metastases Predict Shorter Overall Survival: What Predicts Visceral Metastases? Results from the SEARCH Database. *European urology focus.* 2017;3(4-5):480-486.

24. Rosenbaum PR. Overt bias in observational studies. In: *Observational studies.* Springer; 2002:71-104.

25. Keele L. An overview of rbounds: An R package for Rosenbaum bounds sensitivity analysis with matched data. *White Paper Columbus, OH.* 2010;1:15.

Legends

Table 1: Proportion prescribed enzalutamide and abiraterone acetate respectively, per county and in total.

Table 2: Summary statistics of the AA and ENZ patients for the subset of covariates deemed to be the most important.

Table 3: Standardized difference in percent and variance ratio before and after matching

Figure 1: Description of data on health measures for one randomly chosen enzalutamide-taker

Figure 2: Propensity score overlap, before and after matching

Appendix

Figure S1: The fraction (the number of diagnoses divided by the number of men in the population) with a prostate cancer diagnose.

For peer review only

Table S1: Number of collected first time prescriptions per year after the subsidization, by year of prescription.

Table S2: ICD codes used to specify related diagnoses

Table S3: All variables with description.

Table S4: Estimated factor loadings.

For peer review only

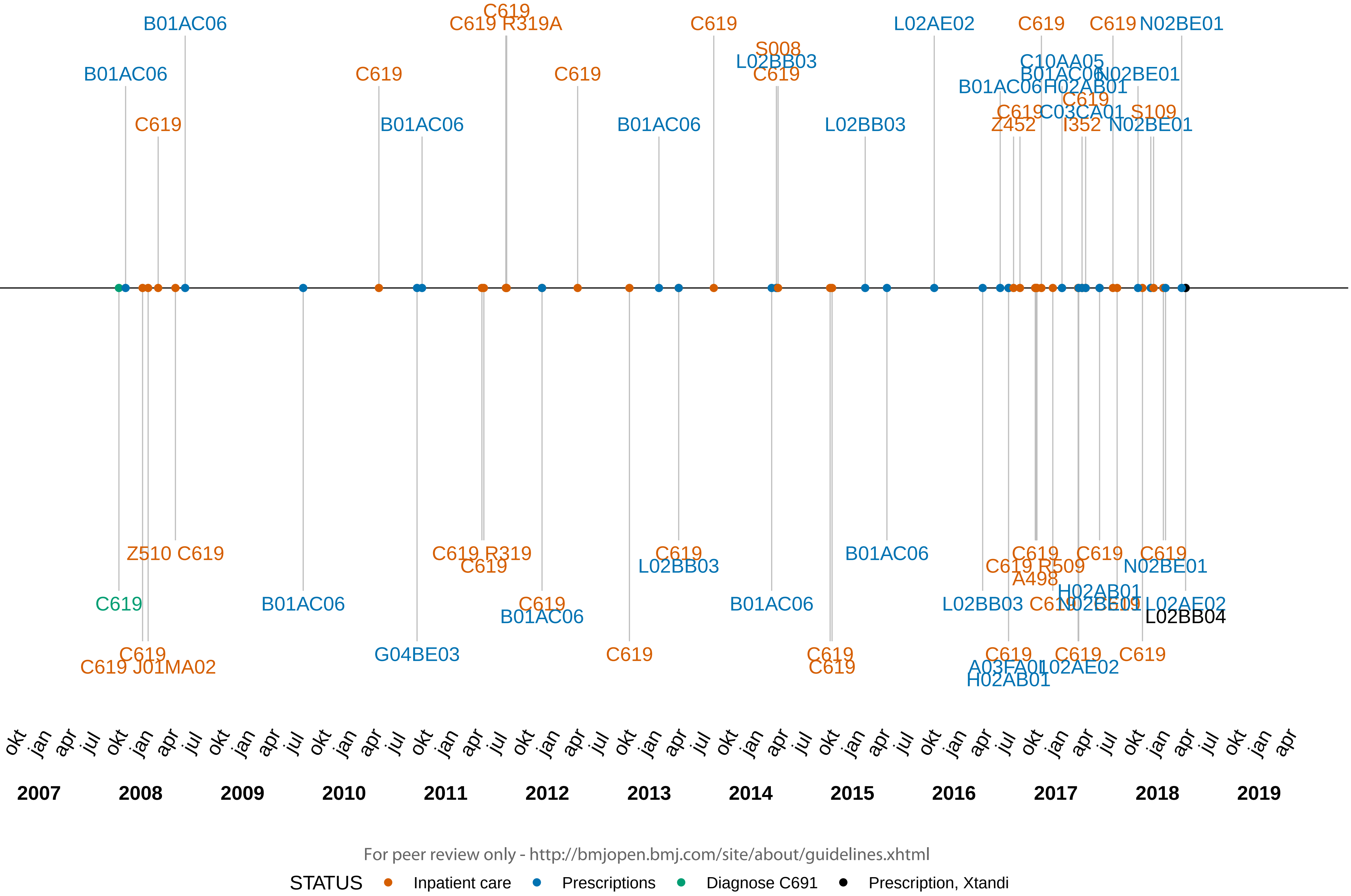
B01AC06
C619
C619 R319A
C619
L02AE02
C619
C619
N02BE01
S008
L02BB03
C10AA05
B01AC06
C619
C619
B01AC06
N02BE01
B01AC06
H02AB01
C619
C619
C03CA01
S109
C619
B01AC06
B01AC06
L02BB03
Z452
I352
N02BE01
Z510 C619
C619 R319
C619
C619
L02BB03
B01AC06
C619
C619 R509
C619
C619
N02BE01
A498
H02AB01
C619
B01AC06
C619
B01AC06
B01AC06
L02BB03
C619
N02BE01
L02AE02
L02BB04
C619
C619 J01MA02
G04BE03
C619
C619
C619
C619
C619
C619
A03FA01
L02AE02
H02AB01
okt jan apr jul
2007
2008
2009
2010
2011
2012
2013
2014
2015
2016
2017
2018
2019
STATUS
Inpatient care
Prescriptions
Diagnose C691
Prescription, Xtandi

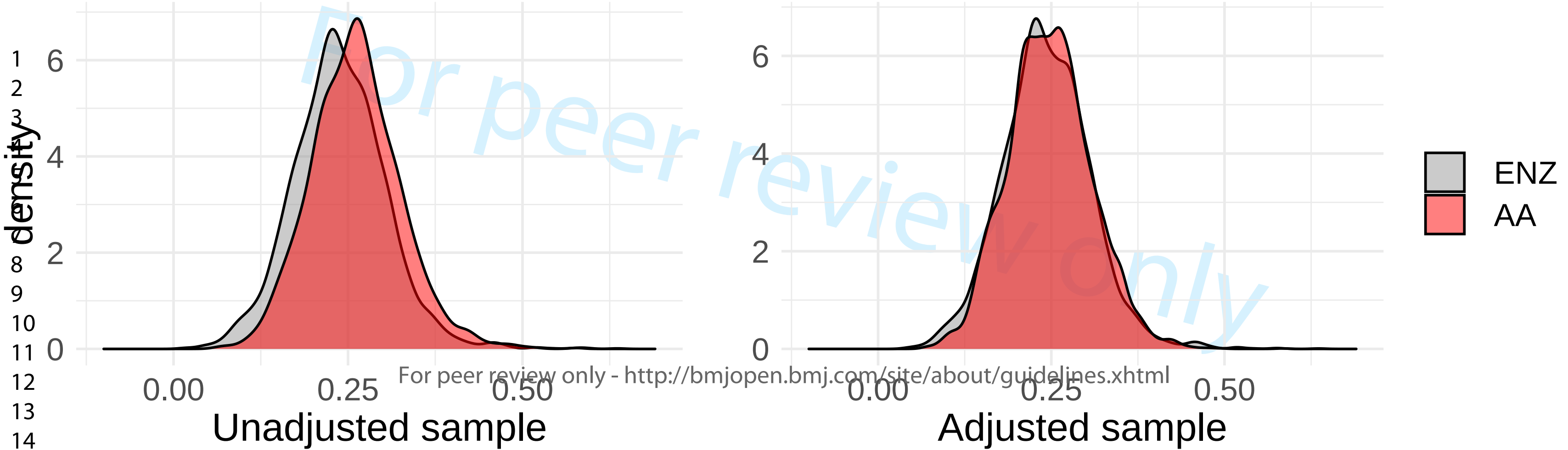


For peer review only

Figure S1: The fraction (the number of diagnoses divided by the number of men in the population) with a prostate cancer diagnose.

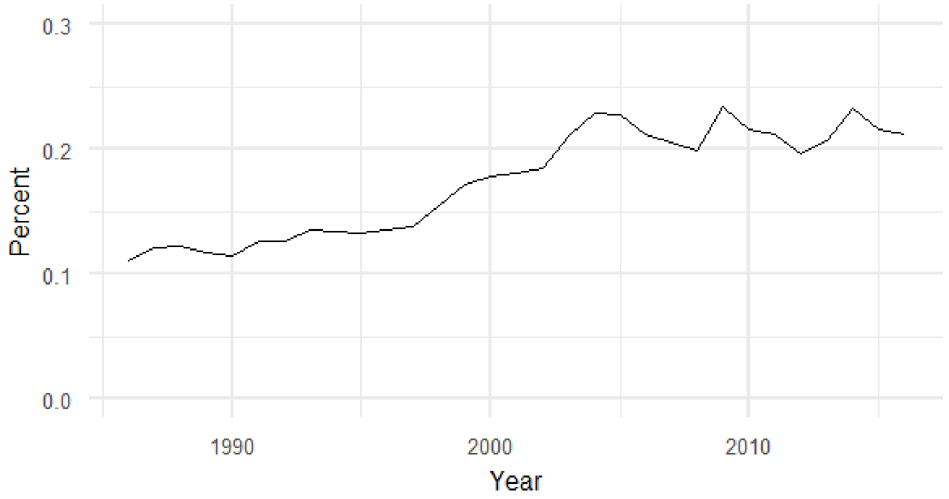


Table S1: Number of collected first time prescriptions per year after the subsidization, by year of prescription.

| Year | Enzalutamide | Abiraterone | Total |
|---|---|---|---|
| 2014 | 0 | 2 | 2 |
| 2015 | 864 | 407 | 1271 |
| 2016 | 1158 | 233 | 1391 |
| 2017 | 1005 | 321 | 1326 |
| 2018 | 454 | 157 | 611 |
| Total | 3481 | 1120 | 4601 |

Note: The reason for the drop in 2018 is that data covers only the period up to June 15 2018.

Table S2: ICD codes used to specify related diagnoses

| Cardiovascular disease | Diabetes | Osteoporosis | Metastases | Malaise and fatigue |
|---|---|---|---|---|
| I21 | E10 | M859 | C77 | R53 |
| I22 | E11 | M810 | C78 | |
| I252 | E12 | M818 | C79 | |
| Z958 | E13 | M819 | | |
| Z959 | E14 | | | |
| I70 | | | | |
| I71 | | | | |

| | | | | |
|---|---|---|---|---|
| I731 | | | | |
| I738 | | | | |
| I739 | | | | |
| I771 | | | | |
| I790 | | | | |
| I792 | | | | |
| K551 | | | | |
| K558 | | | | |
| K559 | | | | |
| I090 | | | | |
| I110 | | | | |
| I13 | | | | |
| I130 | | | | |
| I131 | | | | |
| I132 | | | | |
| I139 | | | | |
| I42 | | | | |
| I420 | | | | |
| I421 | | | | |
| I422 | | | | |
| I423 | | | | |
| I424 | | | | |
| I425 | | | | |
| I426 | | | | |
| I427 | | | | |
| I428 | | | | |
| I429 | | | | |
| I43 | | | | |
| I44 | | | | |
| I45 | | | | |
| I46 | | | | |
| I47 | | | | |
| I48 | | | | |
| I49 | | | | |
| I50 | | | | |
| I51 | | | | |
| R00 | | | | |

For peer review only

Table S3: All variables with description

| | Variable | Description |
|---|---|---|
| | LOPNR | ID serial number |
| | group | Treatment group (group=1 for abiraterone) |
| | lan_namn | County council |
| | h_tot_ov_bfd_i | Indicator of other CVD before diagnosis |
| | h_tot_ov_bw_i | Indicator of other CVD between diagnosis and treatment |
| | d_tot_bfd_i | Indicator of diabetes before diagnosis |
| | d_tot_bw_i | Indicator of diabetes between diagnosis and treatment |
| | o_tot_bfd_i | Indicator of osteoporosis before diagnosis |
| | o_tot_bw_i | Indicator of osteoporosis between diagnosis and treatment |
| | c_tot_bfd_i | Indicator of metastases before diagnosis |
| | c_tot_bw_i | Indicator of metastases between diagnosis and treatment |
| | f_tot_bfd_i | Indicator of fatigue before diagnosis |
| | f_tot_bw_i | Indicator of fatigue between diagnosis and treatment |
| | h_tot_ov_i | Indicator of other CVD before treatment |
| | d_tot | Indicator of diabetes before treatment |
| | o_tot | Indicator of osteoporosis before treatment |
| | c_tot | Indicator of metastases before treatment |
| | i48 | Indicator of atrial fibrillation and flutter before treatment |
| | i21 | Indicator of acute myocardial infarction before treatment |
| | i48_bfd_i | Indicator of atrial fibrillation and flutter before diagnosis |
| | i21_bfd_i | Indicator of acute myocardial infarction before diagnosis |
| | i48_bw_i | Indicator of atrial fibrillation and flutter before diagnosis |
| | i21_bw_i | Indicator of acute myocardial infarction before diagnosis |
| | f_tot | Indicator of fatigue before treatment |
| | h_tot | Indicator of any CVD before treatment |
| | Diff_time | Time between diagnosis and treatment |
| ‘ | bfd_1m | Number of visits, 1 month before diagnosis |
| ‘ | bfd_12m | Number of visits, 12 months before diagnosis |
| ‘ | bfd_60m | Number of visits, 5 years before diagnosis |
| ‘ | afd_1m | Number of visits, 0-1 month after diagnosis |
| ‘ | afd_2m | Number of visits, 1-2 months after diagnosis |
| ‘ | afd_3m | Number of visits, 2-3 months after diagnosis |
| ‘ | bft_1m | Number of visits, 0-1 month before treatment |
| ‘ | bft_2m | Number of visits, 1-2 months before treatment |
| ‘ | bft_3m | Number of visits, 2-3 months before treatment |
| ‘ | bft_4m | Number of visits, 3-4 months before treatment |
| ‘ | bft_5m | Number of visits, 4-5 months before treatment |
| ‘ | bft_12m_tot | Number of visits, 1 year before treatment |
| ‘ | bft_12m_tot_c619 | Number of C61.9 related visits, 1 year before treatment |
| ‘ | h_bfd_12m | Number of heart related visits, 1 year before treatment |

For peer review only

| | | |
|---|---|---|
| ‘ | h_bft_3m | Number of heart related visits, 3 months before treatment |
| ‘ | msv_days_bd | Number of days in inpatient care before diagnosis |
| ‘ | msv_days_c619 | Number of days in inpatient care related to C61.9 |
| ‘ | msv_tot_prop_c619 | Fraction of days in inpatient care related to C61.9 |
| ‘ | msv_days | Days in inpatient care between diagnosis and treatment |
| ‘ | msv_tot_prop | Fraction of days in inpatient care between diagnosis and treatment |
| ‘ | nn | Visits per day between diagnosis and treatment |
| | h_tot_bfd | Visits related to CVD before diagnosis |
| | h_tot_bw | Visits related to CVD between diagnosis and treatment |
| ‘ | d_tot_bfd | Visits related to diabetes before diagnosis |
| ‘ | d_tot_bw | Visits related to diabetes between diagnosis and treatment |
| ‘ | o_tot_bfd | Visits related to osteoporosis before diagnosis |
| ‘ | o_tot_bw | Visits related to osteoporosis between diagnosis and treatment |
| ‘ | c_tot_bfd | Visits related to metastases before diagnosis |
| ‘ | c_tot_bw | Visits related to metastases between diagnosis and treatment |
| ‘ | f_tot_bfd | Visits related to fatigue before diagnosis |
| ‘ | f_tot_bw | Visits related to fatigue between diagnosis and treatment |
| ‘ | i48_bfd | Visits related to atrial fibrillation and flutter before diagnosis |
| ‘ | i21_bfd | Visits related to acute myocardial infarction before diagnosis |
| ‘ | h_tot_ov_bfd | Visits related to other CVD before diagnosis |
| ‘ | i48_bw | Visits related to atrial fibrillation and flutter between diagnosis and treatment |
| ‘ | i21_bw | Visits related to acute myocardial infarction between diagnosis and treatment |
| ‘ | h_tot_ov_bw | Visits related to other CVD between diagnosis and treatment |
| ‘ | q1_t | Number of inpatient care visits between diagnosis and treatment, quartile 1 |
| ‘ | q2_t | Number of inpatient care visits between diagnosis and treatment, quartile 2 |
| ‘ | q3_t | Number of inpatient care visits between diagnosis and treatment, quartile 3 |
| ‘ | q4_t | Number of inpatient care visits between diagnosis and treatment, quartile 4 |
| ‘ | n_heart_kalc | Number of prescriptions of ATC code C08 |
| ‘ | n_heart_beta | Number of prescriptions of ATC code C07 |
| ‘ | n_diab | Number of prescriptions of ATC code A10 |
| | Mort_medel | Historical county specific mortality in prostate cancer |
| | Fodelseland_EU28 | Country of birth |
| | ALDER | Age at treatment |
| | Utbn=ForGymn | Educational level at treatment : less than secondary school |
| | Utbn=Gymn | Educational levelat treatment : secondary school |
| | Utbn=EfterGymn | Educational level at treatment : more than secondary school |
| | Civil | Marital status at treatment (Civil=1 if partner) |
| ‘ | LoneInk.x | Wage income at treatment |

For peer review only

| | | |
|---|---|---|
| ’ | InkFNetto | Income from business at treatment |
| ‘ | ForvErs | Wage income at treatment |
| ’ | KapInk | Capital income at treatment |
| ’ | DispInk04 | Disposable income at treatment |
| ’ | DispInkFam04 | Family disposable income at treatment |
| ‘ | ArbErs | Wage income at treatment |
| ’ | LoneInk_1y | Wage income one year before treatment |
| ‘ | InkFNetto_1y | Income from business one year before treatment |
| ‘ | KapInk_1y | Capital income one year before treatment |
| ‘ | DispInk04_1y | Disposable income one years before treatment |
| ‘ | DispInkFam04_1y | Family disposable income one years before treatment |
| ‘ | ForvErs_1y | Wage income one year before treatment |
| ‘ | LoneInk_2y | Wage income two years before treatment |
| ‘ | InkFNetto_2y | Income from business two years before treatment |
| ‘ | ForvErs_2y | Wage income two years before treatment |
| ‘ | KapInk_2y | Capital income two years before treatment |
| ‘ | DispInk04_2y | Disposable income two years before treatment |
| ‘ | DispInkFam04_2y | Family disposable income two years before treatment |
| ‘ | SjukRe | Sickness compensation at treatment |
| ’ | ArbLos | Unemployment benefits at treatment |
| ’ | ForTid | Early retirement benefit at treatment |
| ‘ | SocInk | Social security benefits at treatment |
| ‘ | SocBidrPersF04 | Social security benefits at treatment |
| ‘ | SocBidrFam | Social security benefits of the family at treatment |
| ‘ | SjukRe_1y | Sickness compensation one year before treatment |
| ‘ | ArbLos_1y | Unemployment benefits one year before treatment |
| ‘ | ForTid_1y | Early retirement benefit one year before treatment |
| ‘ | SocInk_1y | Social security benefits one year before treatment |
| ‘ | SocBidrPersF04_1y | Social security benefits one year before treatment |
| ‘ | SocBidrFam_1y | Social security benefits of the family one year before treatment |
| ‘ | SjukRe_2y | Sickness compensation two years before treatment |
| ‘ | ArbLos_2y | Unemployment benefits two years before treatment |
| ‘ | ForTid_2y | Early retirement benefit two years before treatment |
| ‘ | SocInk_2y | Social security benefits two years before treatment |
| ‘ | SocBidrPersF04_2y | Social security benefits two years before treatment |
| ‘ | SocBidrFam_2y | Social security benefits of the family two years before treatment |
| ‘ | SumAldP03 | Old-age pensions at treatment |
| ‘ | SumTjP | Occupational pensions one year before treatment |
| ‘ | PrivPens | Private pensions two years before treatment |
| ‘ | SumAldP03_1y | Old-age pensions at treatment |
| ‘ | SumTjP_1y | Occupational pensions one year before treatment |
| ‘ | PrivPens_1y | Private pensions two years before treatment |
| ‘ | SumAldP03_2y | Old-age pensions at treatment |
| ‘ | SumTjP_2y | Occupational pensions one year before treatment |

For peer review only - http://bmjopen.bmj.com/site/about/guidelines.xhtml

| | | |
|---|---|---|
| ‘ | PrivPens_2y | Private pensions two years before treatment |
| | ALDER_D | Age at diagnosis |
| | Civil_D | Marital status at diagnosis (Civil=1 if partner) |
| ‘ | LoneInk_D | Wage income at diagnosis |
| ’ | InkFNetto_D | Income from business at diagnosis |
| ’ | KapInk_D | Capital income at diagnosis |
| ’ | DispInk_D | Disposable income at diagnosis |
| ’ | DispInkFam_D | Family disposable income at diagnosis |
| ’ | LoneInk_1y_D | Wage income one year before diagnosis |
| ‘ | InkFNetto_1y_D | Income from business one year before diagnosis |
| ‘ | KapInk_1y_D | Capital income one year before diagnosis |
| ‘ | DispInk_1y_D | Disposable income one year before diagnosis |
| ‘ | DispInkFam_1y_D | Family disposable income one year before diagnosis |
| ‘ | LoneInk_2y_D | Wage income two years before diagnosis |
| ‘ | InkFNetto_2y_D | Income from business two years before diagnosis |
| ‘ | KapInk_2y_D | Capital income two years before diagnosis |
| ‘ | DispInk_2y_D | Disposable two years before diagnosis |
| ‘ | DispInkFam_2y_D | Family disposable income two years before diagnosis |
| ‘ | SjukRe_D | Sickness compensation at diagnosis |
| ’ | ArbLos_D | Unemployment benefits at diagnosis |
| ’ | ForTid_D | Early retirement benefit at diagnosis |
| ‘ | SocInk_D | Social security benefits at diagnosis |
| ‘ | SocBidrPersF_D | Social security benefits at diagnosis |
| ‘ | SocBidrFam_D | Social security benefits of the family at diagnosis |
| ‘ | SjukRe_1y_D | Sickness compensation one year before diagnosis |
| ‘ | ArbLos_1y_D | Unemployment benefits one year before diagnosis |
| ‘ | ForTid_1y_D | Early retirement benefit one year before diagnosis |
| ‘ | SocInk_1y_D | Social security benefits one year before diagnosis |
| ‘ | SocBidrPersF_1y_D | Social security benefits one year before diagnosis |
| ‘ | SocBidrFam_1y_D | Social security benefits of the family one year before diagnosis |
| ‘ | SjukRe_2y_D | Sickness compensation two years before diagnosis |
| ‘ | ArbLos_2y_D | Unemployment benefits two years before diagnosis |
| ‘ | ForTid_2y_D | Early retirement benefit two years before diagnosis |
| ‘ | SocInk_2y_D | Social security benefits two years before diagnosis |
| ‘ | SocBidrPersF_2y_D | Social security benefits two years before diagnosis |
| ‘ | SocBidrFam_2y_D | Social security benefits of the family two years before diagnosis |
| ‘ | AldPens_D | Old-age pensions at diagnosis |
| ‘ | SumTjP_D | Occupational pensions one year before diagnosis |
| ‘ | PrivPens_D | Private pensions two years before diagnosis |
| ‘ | AldPens_1y_D | Old-age pensions at diagnosis |
| ‘ | SumTjP_1y_D | Occupational pensions one year before diagnosis |
| ‘ | PrivPens_1y_D | Private pensions two years before diagnosis |
| ‘ | AldPens_2y_D | Old-age pensions at diagnosis |
| ‘ | SumTjP_2y_D | Occupational pensions one year before diagnosis |

For peer review only

| | | |
|---|---|---|
| ‘ | PrivPens_2y_D | Private pensions two years before diagnosis |

For peer review only

Table S4: Estimated factor loadings

| | F1 | F2 | F3 | F4 | F5 | F6 | F7 | F8 | F9 |
|---|---|---|---|---|---|---|---|---|---|
| SocBidrPers_1y_D | 0.94 | | | | | | | | |
| SocBidrPers_D | 0.93 | | | | | | | | |
| SocBidrFam_D | 0.92 | | | | | | | | |
| SocBidrPers_2y_D | 0.91 | | | | | | | | |
| SocBidrFam_2y_D | 0.90 | | | | | | | | |
| SocBidrFam_1y_D | 0.90 | | | | | | | | |
| SocBidrPersF04_2y | 0.88 | | | | | | | | |
| SocBidrFam_2y | 0.87 | | | | | | | | |
| SocBidrPersF04_1y | 0.87 | | | | | | | | |
| SocBidrFam | 0.86 | | | | | | | | |
| SocBidrPersF04 | 0.85 | | | | | | | | |
| SocBidrFam_1y | 0.84 | | | | | | | | |
| LoneInk_1y | | 0.95 | 0.21 | | | | | | |
| LoneInk.x | | 0.95 | | | | | | | |
| ForvErs_1y | | 0.94 | 0.22 | | | | | | |
| ForvErs | | 0.94 | | | | | | | |
| LoneInk_2y | | 0.92 | 0.25 | | | | | | |
| ForvErs_2y | | 0.91 | 0.27 | | | | | | |
| LoneInk_1y_D | | 0.60 | 0.58 | | | | | -0.37 | |
| LoneInk_D | | 0.59 | 0.56 | | | | | -0.37 | |
| LoneInk_2y_D | | 0.44 | 0.62 | | | | | -0.38 | |
| DispInk04 | | 0.31 | 0.37 | | | | | | |
| DispInk04_1y | | 0.25 | 0.34 | | | | | | |
| DispInk_D | | 0.25 | 0.52 | | | | | | |
| DispInkFam04_1y | | 0.21 | 0.31 | | | | | | |
| SumAldP03 | | -0.30 | | | -0.44 | | | 0.27 | |
| SumAldP03_2y | | -0.31 | | | -0.43 | | | 0.29 | |
| SumAldP03_1y | | -0.32 | | | -0.45 | | | 0.29 | |
| SumTjP_1y | | | 0.87 | | | | | 0.31 | |
| SumTjP | | | 0.85 | | | | | 0.29 | |
| SumTjP_2y | | | 0.84 | | | | | 0.33 | |
| SumTjp_D | | | 0.58 | | | | | 0.70 | |
| SumTjp_1y_D | | | 0.49 | | | | | 0.78 | |
| SumTjp_2y_D | | | 0.39 | | | | | 0.78 | |
| AldPens_D | | | 0.34 | | -0.20 | 0.38 | | 0.75 | |
| DispInk_2y_D | | | 0.32 | | | | | | |
| DispInkFam_D | | | 0.30 | | | | | | |
| DispInk_1y_D | | | 0.30 | | | | | | |
| PrivPens | | | 0.29 | | | 0.59 | | | |
| DispInkFam_2y_D | | | 0.26 | | | | | | |
| DispInkFam04_2y | | | 0.25 | | | | | | |
| DispInkFam_1y_D | | | 0.25 | | | | | | |
| DispInk04_2y | | | 0.25 | | | | | | |
| PrivPens_2y | | | 0.24 | | | 0.92 | | | |
| PrivPens_1y | | | 0.24 | | | 0.91 | | | |
| AldPens_1y_D | | | 0.24 | | | 0.38 | | 0.80 | |

For peer review only

| | | | | | | | |
|---|---|---|---|---|---|---|---|
| DispInkFam04 | 0.23 | | | | | | |
| ForTid_1y_D | | 0.90 | | | | | |
| ForTid_D | | 0.88 | | | | | |
| SocInk_1y_D | | 0.85 | | | | | |
| ForTid_2y_D | | 0.82 | | | | | |
| SocInk_2y_D | | 0.81 | | | | | |
| SocInk_D | | 0.71 | 0.28 | | | | |
| ForTid_2y | | 0.64 | | | | | |
| ForTid_1y | | 0.61 | | | | | |
| ForTid | | 0.57 | | | | | |
| SocInk_2y | | 0.52 | 0.40 | | | | |
| SocInk_1y | | 0.44 | 0.63 | | | | |
| SocInk | | 0.29 | 0.88 | | | | |
| SjukRe_2y_D | | 0.26 | | | | | |
| AldPens_2y_D | | -0.20 | | 0.38 | | 0.79 | |
| ArbErs | | | 0.96 | | | | |
| SjukRe | | | 0.96 | | | | |
| SjukRe_1y | | | 0.74 | | | | |
| SjukRe_2y | | | 0.49 | | | | |
| SjukRe_D | | | 0.45 | | | | |
| nn | | | 0.21 | | 0.42 | | |
| PrivPens_1y_D | | | | 0.94 | | | |
| PrivPens_D | | | | 0.94 | | | |
| PrivPens_2y_D | | | | 0.94 | | | |
| bft_12m_tot | | | | | 0.92 | | |
| bft_12m_tot_c619 | | | | | 0.61 | | |
| bft_3m | | | | | 0.60 | | |
| bft_4m | | | | | 0.59 | | |
| bft_5m | | | | | 0.58 | | |
| bft_2m | | | | | 0.57 | | |
| q4_t | | | | | 0.53 | | |
| bft_1m | | | | | 0.52 | | |
| q3_t | | | | | 0.44 | | |
| c_tot_bw | | | | | 0.35 | | |
| msv_days_c619 | | | | | 0.34 | | |
| q2_t | | | | | 0.34 | | |
| msv_tot_prop | | | | | 0.33 | | |
| bfd_12m | | | | | 0.33 | | |
| bfd_60m | | | | | 0.33 | | |
| msv_days | | | | | 0.33 | | |
| msv_tot_prop_c619 | | | | | 0.30 | | |
| h_bft_3m | | | | | 0.25 | | |
| q1_t | | | | | 0.24 | | |
| h_tot_bw | | | | | 0.22 | | |
| InkFNetto_1y | | | | | | | 0.97 |
| InkFNetto_2y | | | | | | | 0.96 |
| InkFNetto | | | | | | | 0.95 |
| InkFNetto_1y_D | | | | | | | 0.73 |
| InkFNetto_2y_D | | | | | | | 0.71 |
| InkFNetto_D | | | | | | | 0.70 |

For peer review only

For peer review only
